# Witch-hunt

**DOI:** 10.1186/1477-7517-2-3

**Published:** 2005-03-04

**Authors:** Ernest Drucker

**Affiliations:** 1 Montefiore Medical Center, New York, USA; 2 Albert Einstein College of Medicine, New York, USA

## Abstract

Beginning two years ago, the US Dept of Health and Human Services began "special reviews" of all current research grants that involved harm reduction, sex and drugs, and continues its ban on funding of needle exchange. With Bush's second term, the campaign was extended to all US funded international programs that dealt with these issues and populations. And, most recently, the US has again undertaken to dominate the discourse within international organizations charged with drug control and AIDS policies – especially those of the UN. But the international harm reduction and human rights community is fighting back in several important ways, including "An Open Letter to the delegates of the Forty-eighth session of the Commission on Narcotic Drugs (CND) of the UN" prepared by a group of 334 well respected public health experts and human rights advocates, protesting U.S. pressure on the U.N. to withdraw its support from harm reduction. This editorial includes the letter and signatures as well as French, Spanish, and Russian versions of the letter as additional files.

## 

"This is a sharp time, now, a precise time - we live no longer in the dusky afternoon when evil mixed itself with good and befuddled the world. Now, by God's grace, the shining sun is up, and them that fear not light will surely praise it."

Arthur Miller, The Crucible, Act III

"I do not believe that the meaning of our Eighth Amendment, any more than the meaning of other provisions of our Constitution, should be determined by the subjective views of five members of this court and like-minded foreigners"

*Justice Antonin Scalia, in his dissent from the US Supreme Court majority decision barring capital punishment for crimes committed by minors*.

It is indeed "a sharp time" in the US for those of us who agree with "like-minded foreigners". This is especially so regarding matters of drug and AIDS policies based on harm reduction (HR) and public health – decriminalization of drug users, the need for safer injections, low thresholds for access to care, sex education and social supports that work to reduce risk. Today more people are imprisoned for drug use in the US than are incarcerated in the European Union for all crimes.

US conservatives have, in the same lethal moralistic tradition of our Salem witch-hunts of the 1600's and the McCarthy era, effectively obstructed and undermined our HR efforts at home for two decades. But now the Bush administration, emboldened by its re-election and in full warrior mode, has undertaken a newly invigorated global jihad against harm reduction. Americans who support HR are now to be made to feel "foreign", their moral compass, patriotism, and loyalty to "American core – values" placed in doubt.

Parallels to the death penalty are not casual: the failed punitive drug policies of the war on drugs are also official "death sentences", with many more lives lost to them each year than to all the judicially sanctioned executions of all the countries on earth. And, because preventable death is more then an analogy when it comes to public health, we can learn a lot from this Supreme Court case. Justice Anthony M. Kennedy, writing for the courts majority, recognized the "evolving standards of decency" that should shape our judgment of what constitutes a violation of our Constitution's Eighth Amendment and its prohibition against cruel and unusual punishments: "it is fair to say that the United States now stands alone in a world that has turned its face against the juvenile death penalty."

But Justice Scalia, significant as the most likely nominee for Chief Justice with the ailing incumbents imminent departure, saw it differently, the NY Times reporting that he reserved "his strongest dissent for (the majority's) reference to international developments that have left the United States alone in supporting juvenile executions". For while the majority opinion said the court was not bound by foreign developments, "it is proper that we acknowledge the overwhelming weight of international opinion" for its "respected and significant confirmation for our own conclusions", Scalia objected that this position implied that "the views of our own citizens are essentially irrelevant," and had wrongly given "center stage" to the "so-called international community." Assumedly this would be that same " international community" that our Declaration of Independence refers to in its famous opening paragraph where, attempting to justify our nations throwing off British rule, we are told that " a decent respect to the opinions of mankind requires that " we explain our course of action. But as the worlds "sole super- power" (tell China that) it appears that we no longer have to show a decent respect for any other nations opinions about human rights, nor it would seem for the relentlessly insistent biology of HIV.

The US harm maximization drug policies, which violate both human rights and the realities of infectious diseases, are immoral and dangerously misguided – sustained by demagogic politicians and mad moralists now in near absolute power in our country. As surely as capital punishment, these policies mete out death sentences on a massive scale to our most vulnerable citizens. These failed policies account for the continued annual incidence of 40,000 new HIV infections in the US.

Beginning two years ago, the US Dept of Health and Human Services (the parent agency of our National Institutes of Health, which funds most AIDS and drug research in the world) began "special reviews" of all current research grants that involved sex and drugs. Washington re-asserted the drive for mandated "abstinence only" and "faith based" programs, and continues the Federal ban on funding of needle exchange. Don't even bother applying for work on gay sex. With Bush's second term, the campaign was extended to all US funded international programs that dealt with these issues and populations. And, most recently, the US has again undertaken to dominate the discourse within international organizations charged with drug control and AIDS policies – especially those of the UN.

Now the US seeks to impose them as extra judicial capital punishment on the rest of the world. But the international harm reduction and human rights community is not going quietly – it is fighting back in several important ways and we can cite many successes that have already saved hundreds of thousands of lives. Henceforth we will chronicle this struggle in this journal.

As a start we are publishing An Open Letter to the delegates of the Forty-eighth session of the Commission on Narcotic Drugs (CND) of the UN prepared by a group of well respected public health experts and human rights advocates, protesting U.S. pressure on the U.N. to withdraw its support from harm reduction (see Additional file 1). The letter garnered 334 individual and organizational endorsements from fifty-six countries. The organizers of the letter are in the process of sending it to all country missions in Vienna as well as to UNODC Executive Director Antonio Maria Costa and representatives from UNICEF, WHO, UNAIDS, and the UN Office of the High Commissioner for Human Rights.

For more information about this letter contact Jonathan Cohen at Human Rights Watch – cohenj@hrw.org

We at HRJ welcome your views, which can be submitted online (as Comments) at harmreductionjournal.com

• E Drucker PhD is Director, Division of Public Health and Policy Research, Professor of Epidemiology and Social Medicine and Professor of Psychiatry, and Family Medicine at Montefiore Medical Center/Albert Einstein College of Medicine in New York City. Dr. Drucker was a founder of the International Harm Reduction Association and Founding Chairman of Doctors of the World / USA. He is a currently a senior Soros Justice Fellow.

## Appendix

An Open Letter to the delegates of the Forty-eighth session of the Commission on Narcotic Drugs (CND).

In a year when the United Nations Office on Drugs and Crime (UNODC) is chair of the governing body of the UN's Joint Programme on HIV/AIDS (UNAIDS), we write to express concern about U.S. efforts to force a UNODC retreat from support of syringe exchange and other measures proven to contain the spread of HIV among drug users. Injection drug use accounts for the majority of HIV infections in dozens of countries in Asia and the former Soviet Union, including Russia, China, all of Central Asia, and much of Southeast Asia. In most countries outside Africa, the largest number of new infections now occurs among injection drug users. As UNODC director Antonio Maria Costa noted at the July 2004 International AIDS Conference, effective responses to injection driven AIDS epidemics require expanded HIV prevention, including syringe exchange, rather than policies that accelerate HIV infections through widespread and indiscriminate imprisonment.

Unfortunately, recent events suggest that UNODC – under pressure from the United States – is being asked to withdraw support from proven HIV prevention strategies at precisely the moment when increased commitment to measures such as syringe exchange and opiate substitution treatment is needed. It is particularly alarming that the silencing of UNODC is occurring in a year when the agency is chair of UNAIDS' Committee of Co-sponsoring Organizations and in a year when HIV prevention is a focus of thematic debate at the 48th meeting of the CND. Among the events that have particularly heightened our concern are:

* Mr. Costa, who last year expressed support for positive changes in the Russian criminal code, expansion of syringe exchange in countries facing injection driven epidemics and other measures to reduce drug-related harm, has apparently been rebuked by the U.S. State Department. Following a meeting with Robert Charles, U.S. Assistant Secretary for International Narcotics and Law Enforcement Affairs, Mr. Costa pledged to review all UNODC electronic and printed documents for references to "harm reduction" and to be "even more vigilant in the future."

* In Southeast Asia, UNODC has suspended a program that sought reduce drug users' vulnerability to HIV prevention through approaches that emphasized public health and drug users' human rights, rather than punishment.

* Even syringe exchange, affirmed as an effective and essential part of HIV prevention by UNAIDS, WHO, and UN member nations, has become politically unpalatable. A November e-mail from a senior UNODC staff member asked junior staff to "to ensure that references to harm reduction and needle/syringe exchange are avoided in UNODC documents, publications and statements."

We recognize that UNODC is dependent on contributions from donor nations, and that the U.S. is the single largest donor to UN drug control. At the same time, the lives of hundreds of thousands depend on sound, scientific approaches to HIV prevention. Numerous studies, including U.S. government studies, have found that strategies such as syringe exchange and methadone maintenance demonstrably diminish HIV transmission and other health risks. The fact that U.S. delegates declare the evidence in support of syringe exchange "unconvincing," as they did in last year's CND session, should not be allowed to determine the course of the UN drug control and HIV prevention efforts, which are inextricably and essentially linked. Nor should UNODC – a co-sponsor of UNAIDS, and an agency with an essential role to play in the course of the HIV epidemic – be asked to refrain from public statements about needle exchange simply because they do not fall within the realm of what the U.S. deems acceptable.

Strategies that attempt solely to achieve abstinence from drug use do not constitute an acceptable alternative to programs, such as syringe exchange, that help active drug users protect themselves from HIV/AIDS. Experience has shown that "zero tolerance" drug control efforts can have the effect of driving injection drug users underground and away from drug treatment and other health services. This is particularly true where, as in many countries, counter-narcotics efforts lead to false arrest, beatings and extortion by police, prolonged detention without trial, forced drug treatment, disproportionate incarceration in cruel conditions and, in some cases, extrajudicial execution. Programs such as syringe exchange and opiate substitution, by contrast, both prevent HIV infection and can provide a bridge to other health services. Restricting these programs is a blatant infringement of drug users' human right to health.

As you gather this year to debate HIV/AIDS prevention and drug abuse, we respectfully urge you to support syringe exchange, opiate substitution treatment and other harm reduction approaches demonstrated to reduce HIV risk; to affirm the human rights of drug users to health and health services; and to reject efforts to overrule science and tie the hands of those working on the front lines. No less than the future of the HIV epidemic is at stake.

cc: Joint United Nations Programme on HIV/AIDS

World Health Organization

Office of the High Commissioner for Human Rights

International Narcotics Control Board

Organizations and individuals who have signed this letter as of March 1, 2005 are listed in Additional file 1.

For the French version of this Open Letter please see Additional file 2. For the Spanish version of this Open Letter please see Additional file 3. For the Russian version of this Open Letter please see Additional file 4.

## Supplementary Material

Additional File 1 Open Letter to the delegates of the Forty-eighth session of the Commission and other additional information. Click here for file

Additional File 2 French version of the Open Letter Click here for file

Additional File 3 Spanish version of the Open Letter Click here for file

Additional File 4 Russian version of the Open Letter Click here for file

