# Supplementary material for: Witch-hunt
Source: Harm Reduct J. 2005 Mar 4;2:3. doi: 10.1186/1477-7517-2-3 (PMC555579; doi:10.1186/1477-7517-2-3)
Supplement: Additional File 1 — Open Letter to the delegates of the Forty-eighth session of the Commission and other additional information. [file 1477-7517-2-3-S1.doc]

_____________________________________________________________________

The following organizations and individuals have signed this letter as of March 1, 2005:

Organizations by region

Asia

AIZHIXING Institute of Health Education, Beijing, China

Asian Harm Reduction Network (AHRN), Chiang Mai, Thailand

Asia Pacific Rainbow, New Delhi, India

Australian Drug Foundation, Melbourne, Australia

###### Australian Injecting and Illicit Drug Users League, Darlinghurst, Australia

Blue Diamond Society, Kathmandu, Nepal

Burnet Institute, Melbourne, Australia and Yangon, Myanmar

The Centre for Harm Reduction, Macfarlane Burnet Institute for Medical Research & Public Health, Melbourne, Australia

Community Health, Rehabilitation, Education & Awareness (CREA), Dhaka, Bangladesh

Drug Action Committee of the City of Greater Geelong, Victoria, Australia

Family Drug Support, Willoughby, Australia

Health and Development Networks (HDN), Chiang Mai, Thailand

ILGLAW – Asia, New Delhi, India

Lawyers Collective HIV/AIDS Unit, New Delhi, India

Malaysian AIDS Council, Kuala Lumpur, Malaysia

Malaysian Harm Reduction Working Group, Kuala Lumpur, Malaysia

Northern Sydney Central Coast Health, Sydney, Australia

Pinoy Plus Association, Manila, Philippines

RISE, Peshawar, Pakistan

Thai AIDS Treatment Action Group (TTAG), Bangkok, Thailand

Thai Drug Users' Network (TDN), Bangkok, Thailand

Turning Point Alcohol and Drug Centre, Melbourne, Australia

VIVAIDS Inc., Fitzroy, Australia

WartaAIDS, Jakarta, Indonesia

Western Australian Substance Users Association, Bunbury, Australia

Yayasan Spiritia, Jakarta, Indonesia

Africa

AfriCASO: African Council of Aids Service Organization, Dakar, Senegal

Kenya AIDS NGOs Consortium (KANCO), Nairobi, Kenya

Sheryl’s Orphans Children Home, Nairobi, Kenya

UNDP/Kinshasa, Kinshasa, Democratic Republic of Congo

Europe/Central Asia

Abraço—Associação de Apoio a Pessoas Infectadas e Afectadas pelo VIH /SIDA, Lisbon, Portugal

Act Up—Paris, Paris, France

Action Against AIDS Germany, Tubingen, Germany

Actions Traitements, Paris, France

AGIHAS (PLWHA Support group), Riga, Latvia

AIDES NGO, Pantin Cedex, France

AIDS Action Europe, Amsterdam, Netherlands

AIDS Foundation East-West (AFEW), Moscow, Russia

Aids-Hilfe Bonn e. V., Bonn, Germany

AKZEPT E.V.—Bundesverband für akzeptierende Drogenarbeit und humane Drogenpolitik, Berlin, Germany

Amsterdam Institute for Addiction Research (AIAR), Amsterdam, Netherlands

A.N.O., Prague, Czech Republic

Associazione Mastropietro & Co., Turin, Italy

Associazione Nazionale Italiana Lotta AIDS (A.L.A.), Milano, Italy

Blupoint Drug Counselling Centre, Budapest, Hungary

Bremen Institute for Drug Research, Bremen, Germany

CA Odyseus, Bratislava, Slovakia

Center for Interdisciplinary Research on Women and Gender (ZFG), Oldenburg, Germany

Central and Eastern European Harm Reduction Network, Vilnius, Lithuania

The Centre for Research on Drugs and Health Behaviour, Imperial College, United Kingdom

Charitable Foundation "Rehabilitation Center of Drug Addicts "Virtus", Dnipropetrovsk, Ukraine

The Chrysalis Drug Project, Hertford, United Kingdom

Coalition on Vulnerable Population "I Can Live", Vilnius, Lithuania

Community Organization of People Living with HIV & AIDS, Moscow, Russia

Convictus Eesti, Tallinn, Estonia

Cranstoun Drug Services, London, United Kingdom

DIA+LOGS NGO, Support centre for those affected by HIV/AIDS, Riga, Latvia

Die Deutsche Gesellschaft für Suchtmedizin (vorm DGDS) e.V., Hamburg, Germany

DroBeL - Drogenberatung Lehrte e. V., Lehrte, Germany

Drogenberatung e.V., Bielefeld, Germany

Drogprevenciós Alapítvány, Budapest, Hungary

Dublin AIDS Alliance Ltd., Dublin, Ireland

Equal to Equal, Almaty, Kazakhstan

Estima Associação, Leiria, Portugal

Északi Támpont Egyesület, Budapest, Hungary

European AIDS Treatment Group, Brussels, Belgium

European Coalition for Just and Effective Drug Policies (ENCOD), Antwerp, Belgium

European Commission, Coordination of Anti-drugs Policy, Directorate-General Justice, Freedom and Security, Brussels, Belgium

Evangelisches Stadtjugendpfarramt Gera, Gera, Germany

Förderverein interdisziplinärer Sucht- und Drogen-forschung (FISD) e.V., Zentrum für interdisziplinäre Sucht-und drogen-forschung – ZIS, Hamburg, Germany

Freundes- und Förderkreis Suchtkrankenhilfe e.V., Wuppertal, Germany

GAT - Grupo Português de Activistas sobre Tratamentos de VIH/SIDA, Lisbon, Portugal

George House Trust, Manchester, United Kingdom

Grazia Zuffa, Fuoriluogo/Il Manifesto, Rome, Italy

Greater Glasgow Drug Action Team, Glasgow, Scotland

Grup Igia, Barcelona, Spain

Grupo de Trabajo sobre Tratamientos del VIH (gTt), Barcelona, Spain

Health and Social Development Foundation, Sofia, Bulgaria

Health Education Association NGO, Yerevan, Armenia

Helsinki Foundation for Human Rights, Warsaw, Poland

Hemp Seed Association, Budapest, Hungary

HIV Prevention Programme at Kekava, Riga, Latvia

Hope-Sofia, Sofia, Bulgaria

Indro e.V., Münster, Germany

Initiative for Health Foundation, Sofia, Bulgaria

The Initiative of Drug Users' Mutual Support, Vilnius, Lithuania

The Institute for Criminal Policy Research (ICPR), School of Law, King's College, London, United Kingdom

Institut für Sozialpädagogik, Technische Universität Berlin, Berlin, Germany

The International Community of Women Living with HIV/AIDS (ICW), London, United Kingdom

International Harm Reduction Association, London, United Kingdom

Irish Penal Reform Trust, Dublin, Ireland

Itaca Europe, Rome, Italy

Italian League for the fight against AIDS - Center for Human Rights and Public Health (LILA CEDIUS ONLUS), Milan, Italy

JES e.V., Bielefeld and Bremen, Germany

John Mordaunt Trust, London, United Kingdom

Mainline Foundation, Amsterdam, Netherlands

Malopolskie Stowarzyszenie PROBACJA (PROBACJA Association), Krakow, Poland

MARATON, Warsaw, Poland

Methadone Alliance, London, United Kingdom

MODUS VIVENDI, Prévention du sida et réduction des risques pour usagers de drogues, Brussels, Belgium

Monar Krakow Drugs Project, Krakow, Poland

MONAR, Pulawy, Poland

National AIDS Trust, London, United Kingdom

Netherlands Drug Policy Foundation (SDB), Haarlem, Netherlands

NGO TRUST, Skopje, Macedonia

Osservatorio Italiano sull'Azione Globale contro l'AIDS, Rome, Italy

PASSAGE, Skopje, Macedonia

Plymouth Drug and Alcohol Action Team, Plymouth, United Kingdom

Quest for Quality, Amsterdam, Netherlands

REFORM, Drug Policy Interest Group, Essex, United Kingdom

RFSU, The Swedish Association for Sexuality Education, Stockholm, Sweden

Romanian Association against AIDS (ARAS), Bucharest, Romania

Romanian Harm Reduction Network (RHRN), Bucharest, Romania

Russian Harm Reduction Network, Moscow, Russia

SANANIM, Prague, Czech Republic

Sensoa (Flemish Centre for Expertise and Services on Sexual Health and HIV), Antwerp, Belgium

SIDACTION – International Programs, Paris, France

SIDA STUDI, Barcelona, Spain

SignPost Forth Valley, Stirling, Scotland

Social AIDS Committee, Warsaw, Poland

SOMA – Associação Portuguesa Anti-proibicionista, Lisbon, Portugal

Steunpunt Druggebruikers, Antwerp, Belgium

T3E [UK] Ltd., London, United Kingdom

Transform Drug Policy Foundation, Bristol, United Kingdom

Transnational Institute (TNI) Drug and Democracy Programme, Amsterdam, Netherlands

Tver AIDS Center, Tver, Russia

Tver Center of Drugs Addiction Treatment, Tver, Russia

United Kingdom Harm Reduction Alliance Eccles, Kent, United Kingdom

Women for Women's Human Rights (WWHR) - New Ways, Istanbul, Turkey

World AIDS Campaign, Amsterdam, The Netherlands

Zentrum für interdisziplinäre Frauen- und Geschlechterforschung – ZFG, Carl von Ossietzky Universität Oldenburg, Oldenburg, Germany

Zentrum Suchtmedizin Klinikum Wahrendorff, Sehnde, Germany

Latin America

Argentinean Harm Reduction Association (ARDA), Buenos Aires, Argentina

Argentinean Harm Reduction Network (REDARD), Buenos Aires, Argentina

Asociación Civil “Convivencia” de Personas Viviendo y Conviviendo con VIH/SIDA, Buenos Aires, Argentina

Brazilian Drug Users Network, Recife, Brazil

Fundación para Estudio e Investigación de la Mujer. Buenos Aires, Argentina

HUMANAR, Huritaba, Brazil

Intercambios Asociación Civil, Buenos Aires, Argentina

International Women Aids Caucus, Buenos Aires, Argentina

LACCASO - Latin American and Caribbean Council of AIDS Service Organizations, Caracas, Venezuela

Mujer y Salud en Uruguay (MYSU), Montevideo, Uruguay

ONG INTERPARES, Parana Entre Rios, Argentina

Parana Drug Users Network, Curitiba, Brazil

Programa Alter-Acciones, El Abrojo – Instituto de Educación Popular, Montevideo, Uruguay

REDUC, Brazilian Harm Reduction Network, São Paulo, Brazil

RELARD, Latin American Harm Reduction Network, Curitiba, Brazil

Middle East/North Africa

Iranian National Center for Addiction Studies (INCAS), Tehran, Iran

The Moroccan Association for the Fight Against AIDS, Marrakesh, Morocco

Persepolis Harm Reduction NGO, Tehran, Iran

Persia+, UNDP GIPA Programme, Tehran, Iran

North America

Access Works! Harm Reduction Services, Minneapolis, MN, USA

Act Up—East Bay, Oakland, CA, USA

Advocates for Recovery Through Medicine, Connecticut Chapter, New London, CT, USA

After Hours Project, Inc., Brooklyn, NY, USA

AIDS Foundation of Chicago, Chicago, IL, USA

AIDS Project Los Angeles, Los Angeles, CA, USA

AIDS Treatment Data Network, New York, NY, USA

American Academy of HIV Medicine, Washington DC, USA

American Foundation for AIDS Research, Washington DC, USA

ARC International, Ottawa, Ontario, Canada

Canadian Harm Reduction Network, Toronto, Ontario, Canada

Canadian HIV/AIDS Legal Network, Montreal, Quebec, Canada

Center for Health and Gender Equity (CHANGE), Takoma Park, MD, USA

Center for Human Rights and Public Health, Johns Hopkins Bloomberg School of Public Health, Baltimore, MD, USA

CHAMP (Community HIV/AIDS Mobilization Project), New York, NY, USA

Chicago Recovery Alliance, Chicago, IL, USA

CitiWide Harm Reduction, Bronx, NY, USA

Drug Overdose Prevention & Education Project, San Francisco, CA, USA

DrugSense, Irvine, CA, USA

Exponents, New York, NY, USA

Foundation for Integrative AIDS Research (FIAR), Brooklyn, NY, USA

Gay Men's Health Crisis, New York, NY, USA

Global AIDS Alliance, Washington DC, USA

Harm Reduction Coalition, New York, NY, USA

Harm Reduction Project, Denver and Salt Lake City, UT, USA

Health GAP (Global Access Project), New York, NY, USA

HIV Advocacy Council of Oregon and SW Washington, Portland, OR, USA

HIV Resource Center, Roseburg Risk Reduction, Roseburg, OR, USA

Housing Works, Inc., New York, NY, USA

Human Rights Watch, New York, NY, USA

International Antiprohibitionist League, New York, NY, USA and Rome, Italy

International Center for Advancement of Addiction Treatment, Baron Edmond de Rothschild Chemical Dependency Institute of Beth Israel Medical Center, New York, NY, USA

International Council of AIDS Service Organizations (ICASO), Toronto, Ontario, Canada

International Foundation for Alternative Research in AIDS (IFARA), Portland, OR, USA

International Gay and Lesbian Human Rights Commission, New York, NY, USA

International Women's Health Coalition, New York, NY, USA

Lower East Side Harm Reduction Center, New York, NY, USA

Montefiore Medical Center, Division of Public Health and Policy Research, Bronx, NY, USA

NAMA-Norcal, Santa Cruz, CA, USA

National Association of People with AIDS (NAPWA), Silver Spring, MD, USA

National Association for Victims of Transfusion-Acquired AIDS, Bethesda, MD, USA

The New York Academy of Medicine, New York, NY, USA

Open Society Institute, New York, NY, USA

Physicians for Human Rights, Washington, DC, USA

Positive Health Project, Inc., New York, NY, USA

Prevention Point Pittsburgh, Pittsburgh, PA, USA

TAG, Treatment Action Group, New York, NY, USA

Unified Networkers of Drug Users Nationally, Kingston, Ontario, Canada

Individual Signatories

Vittorio Agnoletto, Scientific Mgr of LILA CEDIUS, Member of the European

Parliament, GUE/NGL Group, Italy

Waheed Ahmad, Advocate High Court, Legal Secretary: World Asian Workers Organisation, Lahore, Pakistan

Joan Anderson, HIV/AIDS community Consultant and volunteer in Toronto, Ontario, Canada

Guillermo R. Aureano, Ph.D., Chercheur associé, Groupe d'étude et de recherche sur la sécurité internationale (GERSI), Département de science politique, Université de Montréal, Montréal, Quebec, Canada

Brambillaschi Barbara, L.I.L.A. - Lega Italiana per la Lotta contro l'AIDS-sede di Como, Italy

Trude Bennett, The University of North Carolina at Chapel Hill, School of Public Health, Chapel Hill, NC, USA

Dennis Berg, Ph.D., Professor of Sociology, California State University, Fullerton, CA, USA

Marie-Andrée Bertrand, Professor emeritus, University of Montreal, Criminology, Montréal, Quebec, Canada

Suzanne Bessoir, Harm Reduction Specialist, AIDS Service Center NYC, New York, NY, USA

Calum Blair, Harm Reduction worker, SignPost Forth Valley, Stirling, Scotland

Simon Boerboom, Psychiatrist, Amsterdam, Netherlands

Beth M. Bouloukos, Ithaca, New York, NY, USA

Scott Burris, James E. Beasley Professor of Law, Temple University Beasley School of Law, Philadelphia, PA, USA

Ryan Borgen, Student Global AIDS Campaign, Northeastern University School of Law, Boston, MD, USA

Jill Britton, London, United Kingdom

Damon Brogan, Manager, VIVAIDS, Melbourne, Australia

Benjamin Bynum, Fikelela AIDS Project, Cape Town, South Africa

Vincenzo Caracciolo, Associazione P24 Lila Livorno, Livorno, Italy

Craig Carmichael, The University of Queensland, Brisbane, Australia

Paul Causey, HIV/AIDS Program Consultant, Bangkok, Thailand

Jennifer Chapman, MPH, Project Manager/Research Coordinator, Center for Clinical, Epidemiology & Biostatistics, University of Pennsylvania, School of Medicine, Philadelphia, PA, USA

George K. Clarke, Volunteer CT Director, CMA, New London, CT, USA

Shelley Cogger, Research Assistant, Post Release Experience of Prisoners in Queensland (PREP-Q), Queensland Alcohol and Drug, Research and Education Centre (QADREC), University of Queensland, Australia

Franco Corleone, Forum Droghe, Rome, Italy

Melissa Dent, Community Development Worker, Melbourne, Australia

Tamara Desiatov, Koondoola, Western Australia

Kate Dickie, Annah Pickering, Managers, Auckland Branch of the New Zealand Prostitutes Collective, Auckland, New Zealand

Dr. Gyaw Htet Doe, Senior Consultant Psychiatrist, Taunggyi Drug Treatment Hospital, Taunggyi, Myanmar

Ms J Dowling, Auburn, NSW, Australia

Andy Dudley, Addaction, Derby, United Kingdom

Carolina Pecheny Durozier, Paris, France

Brian R. Edlin, M.D., Associate Professor of Medicine and Public Health, Center for the Study of Hepatitis C, Weill Medical College of Cornell University, New York, NY, USA

Maria Fotopoulou, Ph.D. student, Imperial College, University of London, London, United Kingdom

Mayada Youssef Fox, staff member, HIV/AIDS Department, WHO HQ, Geneva, Switzerland

Sandra Fox, former staff, Harm Reduction sector, Melbourne, Australia

Jonathan Freedlander, Baltimore, MD, USA

Liliana Gherman, MD, Program Director, Public Health Program, Soros Foundation, Chisinau, Moldova

Cees Goos, honorary consultant Anton Proksch Institut, former WHO staff, Vienna, Austria

Chris W. Green, Jakarta, Indonesia

Heath Greville (individual support), Department of Health, Perth, Western Australia

Patrick Griffiths, PhD candidate, Contemporary Globalisation and HIV/AIDS in Vietnam, RMIT University, Melbourne, Australia

Revd Dr Ian T Guy, General Practitioner, Fulcrum Medical Practice, Middlesbrough, United Kingdom

Bianca L. Guzman, Ph.D., CHOICES Director of Research, La Puente, CA, USA

Fiona Hale, International Network Manager, ICW, London, United Kingdom

Jen Hall, Information and Referral (Duty) Worker, Inner South Community Health Service, Prahran, Australia

Helena Hansen, MD-Ph.D. Candidate, Yale University School of Medicine, New Haven, CT, USA

Paul Hardacre, Training Officer, Asian Harm Reduction Network (AHRN), Chiang Mai, Thailand

Frank Harding, Ayer's Cliff, Quebec, Canada

Andy Hart, Vendafit Pty Ltd, Sydney, Australia

Paul J. von Hartmann, Project P.E.A.C.E., Planet Ecology Advancing Conscious Economics, Santa Rosa, CA, USA

Robert Heimer, Ph. D., Associate Professor, Department of Epidemiology and Public Health Center for Interdisciplinary Research on AIDS Yale University School of, New Haven, CT, USA

Beatriz Acevedo Holguin, Researcher on International Drug Policy, Hull, United Kingdom

Lital Hollander, Research Manager, ESMAN Medical Consulting, Milano, Italy

Danny Holness, Peer Education Officer, RaveSafe, Victoria, Australia

Daniel E. Hood, Ph.D., Department of Criminal Justice/Security Systems, State University of New York at Farmingdale, Farmingdale, New York, NY, USA

Annabelle Horton, UN Office of Drugs and Crime, Bangkok, Thailand.

Neil Hunt, Senior Research Associate, University of Kent; Honorary Research

Fellow, Centre for Research on Drugs and Health Behaviour, Imperial College,

London; Director, UK Harm Reduction Alliance Eccles, Kent, United Kingdom

Elena Jeffreys, Scarlet Alliance, Australias Sex Worker Association, Canberra Act, Australia

McKinzie McClay Jernberg, Minneapolis, MN, USA

Debbie Johnson, Macclesfield, Cheshire, United Kingdom

P. R. W. Kendall, MBBS, MSc, FRCPC, Provincial Health Officer, Ministry of Health Services, Victoria, British Columbia, Canada

Joseph Kim, Young Drug Users Peer Education Officer, VIVAIDS (Victorian Drug User

Organisation), Melbourne, Australia

Dr Stuart A. Kinner, Lecturer, Queensland Alcohol and Drug, Research and Education Centre (QADREC), Queensland, Australia

Debbie Kocziban, Addaction, Plymouth Devon, United Kingdom

Nicky Kupfer, Alcohol Tobacco and other drugs service, Sydney, Australia

Heather La Faye LPN, Medicine cures sickness,all the world is medicine, what is the self?, Recovery Resource Center, Minneapolis, MN, USA

Susana Lambrechts, Esteban Echeverría, Argentina

Deb Lapthorne, Director of Public Health, Plymouth Primary Care Trust/ Plymouth City Council, Plymouth, United Kingdom

Fiona Leibrick, Lecturer: Health Sciences (Alcohol & Other Drug Studies) / Registered Psychologist, Charles Darwin University, Darwin, Australia

Sarah Lippek, AIDS Center of Queens County, Long Island City, NY, USA

Deirdre Love, Senior Health Promotion Specialist HIV Positive People, Health First, London, United Kingdom

Wendy Loxley, Associate Professor, National Drug Research Institute, Curtin University of Technology, Bentley, West Australia

Devon MacFarlane, Vancouver, British Columbia, Canada

Catherine Mackenzie, St. Catharines, Ontario, Canada

Jan Chrostek Maj MD, Rydygier's Hospital, Krakow, Poland

Paolo La Marca, Project manager (Harm reduction & training), LILA CEDIUS (Italian league fight against AIDS), Milano, Italy

David Martin, Program Medical Officer, Health Canada, Vancouver, British Columbia, Canada

Phyra M. McCandless, Johns Hopkins School of Public Health, Baltimore, MD, USA

Rachel McLean, MPH Candidate, Johns Hopkins University Bloomberg School of Public Health, Baltimore, MD, USA

Belinda McNair, Senior Project Officer-City Drug Safety Plan, City of Melbourne, Melbourne, Australia

Cristina Menoyo, Secretaría del Plan Nacional sobre el Sida, Ministerio de Sanidad y Consumo, Madrid. España

Maria Luisa Milesi, Esteban Echeverría, Argentina

Dr Peter Miller, Senior Clinical Research Worker. National Addiction Centre (Maudsley Hospital/Institute of Psychiatry). King's College London, London, United Kingdom

PhDr. Michal Miovsky, Ph.D., Institute of psychology, Academy of Sciences of the Czech Republic, Prague, Czech Republic

Andrew Moss Ph.D, Professor of Epidemiology and Medicine, University of California, San Francisco, CA, USA

Marjorie "Mo" Mowlam, Kent, United Kingdom

Sam Muller, Regional Field Coordinator, Asia Regional HIV/AIDS Project, "An Australian Government Initiative", Ha Noi, Viet Nam

Roshan das Nair, Queen's Medical Centre, Nottingham, England

Dr. Russell Newcombe, Senior Lecturer in Drug Use & Addiction, School of Psychology, Faculty of Science, Liverpool John Moore's University, Liverpool, England

Dr Michael O'Dwyer, Senior Health Adviser, DFID South East Asia, Bangkok, Thailand.

Patrick O'Gorman, Consultant with Asian Harm Reduction Network and European Expert with UNDP led BUMAD and SCAD programmes, Nottingham, United Kingdom

James M. Oleske, MD, MPH, François-Xavier Bagnoud Professor of Pediatrics, Director, Division of Pulmonary, Allergy, Immunology & Infectious Diseases, Department of Pediatrics, New Jersey Medical School, Newark, NJ, Untied States

Hilgunn Olsen, SIRUS Norwegian Institute for Alcohol and Drug Research, Oslo, Norway

Kim Pate, Canadian Association of Elizabeth Fry Societies (CAEFS), Ottawa, Ontario, Canada

Mario Pecheny, Instituto Gino Germani, Universidad de Buenos Aires – CONICET, Buenos Aires, Argentina

Jia Ping, Beijing Aizhixing institute of health education, China

Dr.Hayley Pinto, Norfolk and Waveny Mental Health, Partnership NHS Trust, Norwich, Norfolk, United Kingdom

Robert Power PhD, Reader in Health & Social Sciences Research, Centre for Sexual Health & HIV Research, Department of Primary Care & Population Sciences, Royal Free & University College Medical School, Mortimer Market, London, United Kingdom

Dianne Proctor, Reproductive Health Activist, (Previously CEO of AHRA, now retired), Canberra, Australia

Kenn Quayle, i2i Peer Support, Gibsons, British Columbia, Canada

Pedro Ratis e Silva, DINAMO, São Paulo, Brazil

Melissa Raven, Lecturer, Coordinator, Drugs and Public Health, Department of Public Health, Flinders University, Bedford Park, Australia

Emran M. Razzaghi, M.D., M.P.H., Ass Professor of Psychiatry, Tehran University of Medical Sciences 2004-5 World Fellow, Yale University, New Haven, CT, USA

Thomas Timon Reichl, Bonn, Germany

Josiah D. Rich, M.D., M.P.H., Associate Professor of Medicine and Community Health, Brown University, Providence, RI, USA

Jane Richman, Substance Misuse Worker, United Kingdom

Kay Roberts, Glasgow, United Kingdom

Xavier Majó Roca, Programme on Drug Abuse, Department of Health. Autonomous, Government of Catalonia, Barcelona, Catalonia-Spain

Allan Rosenfield, MD, Mailman School of Public Health, Columbia University, New York, NY, USA

Rainer Rotthoff, Pelangi Communitry Foundation, Kuala Lumpur, Malaysia

Cliff Seaward, drug worker, United Kingdom

Gerard M. Schippers, Ph.D., Professor of Addictive Behaviors and Treatment Evaluation, Amsterdam Institute for Addiction Research (AIAR), Amsterdam, The Netherlands

Susan G. Sherman, PhD, MPH, Assistant Professor, Johns Hopkins Bloomberg School of Public Health, Baltimore, MD, USA

Ram Singh Gurung, an Ex-drug user, Advocacy coordinator, Naya Goreto, Kathmandu Nepal

Alfred Sommer, Dean and Professor, Johns Hopkins Bloomberg School of Public Health, Baltimore, MD, USA

Dr Mónica Suárez Cardona, Madrid, Spain

Brent Taylor, UNDUN, Kingston, Ontario, Canada

Dr. Gerald Thomas, Ottawa, Ontario, Canada

Juan Gabriel Tokatlian, Director Political Science and International Relations, Universidad de San Andres, Argentina

Bruce G. Trigg, MD, Medical Director, Sexually Transmitted Diseases Program, Department of Health, NM, USA

Pervaiz Tufail, Program Manager, AMAL Human Development Network, Islamabad, Pakistan

Azmi bin Uda, "Positive Muslim", Marang,Kuantan and Kuala Lumpur, Malaysia

Jamie Uhrig, Consultant in HIV Prevention and Care, Chiang Mai, Thailand

Kenneth A. Vail, M.P.H, M.A., Tenderloin AIDS Resource Center, San Francisco, CA, USA

Félix Vanderstricht, Aquadev NGO, Tools & Strategy Dpt., Brussels, Belgium

Camila Vega, Consultant, Regional Cooperative Mechanism to Monitor and Execute the ACCORD Plan of Action, United Nations Office on Drugs and Crime, Regional Centre for East Asia and the Pacific, Bangkok, Thailand

Miguel García Villanueva, Head of the Medical Services of the Prison of Pamplona, Spain

David Vlahov, Ph.D., New York, NY, USA

Jennifer Whittall, RhythmicPrinting, Victoria, British Columbia, Canada

Karen Willey, Fulcrum Medical Practice, Middlesbrough, England

Dr. Alex Wodak, Director, Alcohol and Drug Service, St. Vincent's Hospital, Sydney, Australia

Liron B. Wolff, LMSW, New York, NY, USA

Yang Yang, MPH, Yale University of Public Health, New Haven, CT, USA

Yuanyin, The Central University for Nationalities, China

Tomas Zabransky, M.D., Ph.D., Institute for Epidemiology, Public Health and Hygiene, Palacky University Olomouc, Czech Republic

------------------------------------------------------------------------http://hrw.org/english/docs/2005/03/02/global10244.htm

©

February 28, 2005

PAGE ONE

DOW JONES REPRINTS

This copy is for your personal, non-commercial use only. To order presentation-ready copies for distribution to your colleagues, clients or customers, use the Order Reprints tool at the bottom of any article or visit:

www.djreprints.com.

• See a sample reprint in PDF format.

• Order a reprint of this article now.

MAKING PROGRESS

• AIDS Scientists Cite Modest Gains3

Bush Ties Money

For AIDS Work

To a Policy Pledge

By MICHAEL M. PHILLIPS

Staff Reporter of THE WALL STREET JOURNAL

*February 28, 2005; Page A3*

WASHINGTON -- The Bush administration is barring private American AIDS organizations from winning federal grants to provide health services overseas unless they pledge their opposition to prostitution, as part of a broader Republican effort in recent weeks to apply conservative values to foreign-assistance programs.

The White House move comes as Republican lawmakers have been pressing the administration to cut off funds to private organizations that encourage clean-needle programs overseas for intravenous drug users -- a group at the center of the AIDS epidemic in Central Asia and other areas. Some also are pressing to ban federal funding of all AIDS organizations that fail to accept the president's social agenda on such issues as sexual abstinence and drug abuse.

At stake are billions of dollars in U.S. funds that private health organizations working in the developing world spend on AIDS programs (See related article1.)

Administration officials recently started requiring U.S. AIDS groups seeking federal grants as support for their overseas programs to sign a pledge publicly opposing prostitution.

"There is conservative support" for AIDS programs, said Sen. Sam Brownback, a Kansas Republican. "But there are areas of concern...that risk the continued support from a number of conservative members and conservative groups."

Many AIDS organizations are reluctant to issue a statement condemning prostitution because they work closely with prostitutes on health initiatives such as distributing condoms. The groups say such official stigmatization would increase the women's isolation, making it harder for them to receive AIDS prevention and treatment services. Many nongovernmental organizations in the AIDS field are critical of the administration moves.

"This is another salvo in the campaign that the administration and its fellow conservatives are undertaking to create more and more litmus tests and blacklists of those they're willing to do business with," said Susan Cohen, director of government affairs for the Alan Guttmacher Institute, a private think tank that does research on sexual and reproductive health and favors abortion rights.

The dispute marks an escalation in the decades-long debate over attaching moral strings to U.S. foreign assistance. Until now, that battle has centered largely on whether U.S. aid should go to groups providing abortion counseling and services overseas.

The new policy shift regarding prostitution stems from two 2003 laws, one applying to AIDS grants and the other to sex trafficking, which involves luring or forcing individuals into prostitution. The Bush administration had previously applied the requirement only to overseas groups because the Justice Department initially advised that it would be an unconstitutional violation of free speech to demand that American grant applicants support Mr. Bush's policy. But the Justice Department reversed itself last fall.

The charged debate over morality and AIDS programs has drawn new fuel recently from the practice known in the AIDS field as "harm reduction." Many AIDS groups -- some of them considered liberal on social issues -- say the best way to limit the disease is to acknowledge that some people inevitably engage in risky behavior -- intravenous drug use, prostitution or multipartner sex, for example -- and health workers should try to both discourage those activities and make them less dangerous.

Some conservative groups, on the other hand, urge a just-say-no approach, arguing that making prostitution and intravenous drug use less risky encourages people to engage in them. At a recent congressional hearing, John Walters, the director of the Office of National Drug Control Policy, said, "We have been pretty aggressive with international bodies that have...drifted toward harm reduction, more aggressive than I believe others have been in the past."

Mr. Bush, who has made AIDS prevention and treatment a centerpiece of his effort to convey a compassionate side to his conservatism, asked Congress for $3.2 billion for international HIV programs for fiscal 2006. Most such spending is channeled through the U.S. Agency for International Development and the Department of Health and Human Services to private organizations and other health groups working in developing nations.

The new strictures from the White House and Congress match proposals of various conservative religious groups that claim credit for helping the president win re-election. Some are now for the first time applying for such grant money.

Janice Crouse, a senior fellow at Concerned Women for America, an evangelical lobbying and advocacy group, says left-leaning groups have long dominated international AIDS programs, and the changes pursued by the administration and Congress aim to redress that imbalance.

Ms. Crouse describes the dominant side as a "connected inside group of people who are mostly liberal," and says, "They have large staffs, they have experts in grant writing, and they've had almost exclusive access to government and foundation funding."

Until last year, CWA had never applied for government funding or ventured across the line between advocacy and hands-on operations. In November, however, the group won a $113,000 State Department grant to teach Mexican church and community leaders to combat sex trafficking. The group hopes to apply for more money to help the Mexicans set up hotlines and shelters for victims of sex trafficking.

Some health groups charge that the administration and Republicans are imposing their social agenda on a medical crisis. "Social conservatives inside and outside this administration are going way beyond trying to transform what the government funds to focusing on who the government funds," Ms. Cohen said.

A major target of congressional Republicans is an institute founded by billionaire investor George Soros, who spent millions of dollars during last year's presidential campaign trying to defeat Mr. Bush. Mr. Soros's Open Society Institute supports programs that allow heroin addicts in the former Soviet bloc to swap dirty syringes for clean ones in order to limit the spread of HIV. The group receives some federal funds, though Mr. Soros's aides say that money isn't applied to needle-exchange programs.

Marc Wheat, chief counsel to the Subcommittee on Criminal Justice, Drug Policy and Human Resources, says his boss, Indiana Republican Rep. Mark Souder, began investigating Mr. Soros's group before Mr. Soros became involved in the presidential campaign. "We've been concerned about what the Open Society Institute has been doing for a while," Mr. Wheat said.

Under the new antiprostitution requirement, even organizations whose prevention and treatment programs for AIDS have nothing to do with prostitutes must now certify in writing their acceptance of the pledge or face a funding ban.

"If you're trying to go after the spread of HIV, it is not inconsistent to be concerned about issues of prostitution," said Kent Hill, USAID's acting assistant administrator for global health.

Some private organizations expressed dismay at the new policy. "I'm sure there are good intentions motivating the implementation of this policy, but...we feel very concerned that this will fuel stigma against sex workers," said Geeta Rao Gupta, president of the International Center for Research on Women.

U.S. officials say some AIDS grant applicants have signed the pledge, but the administration won't identify them.

While the administration has focused on prostitution, Republicans in Congress are working to yank federal funding from private groups that advocate or discuss clean-needle exchange programs. Leaders of that effort include Reps. Souder and Tom Davis, the Virginia Republican. Sen. Brownback laid out his goals in a strategy memo for allies this month that called for a ban on USAID grants to organizations that don't fully support the president's views on issues ranging from drug use to sexual abstinence.

"How many lives have been saved from this totally preventable disease by the 'disease control' efforts of these longstanding and aggressive family planners, drug legalizers and pro-prostitution groups?" the memo asked rhetorically.

The Brownback memo singled out Population Services International, a Washington-based nonprofit organization involved in family planning and AIDS work abroad, for sponsoring a sexually suggestive condom ad on Kenyan television, even though the ad itself wasn't funded by the U.S. government. PSI says that sexually provocative ads are the most effective in getting people's attention and persuading them to practice safer sex.

The memo also accused groups associated with Mr. Soros of using USAID funds to hand out clean needles in Eastern Europe and Asia. USAID policy forbids using federal money to finance needle exchanges.

Aryeh Neier, president of Mr. Soros's Open Society Institute, said it doesn't take a position on drug legalization or use federal money to finance its needle-exchange programs in Central Asia. The institute uses some USAID money to discourage drug use, but is largely funded by $400 million a year from Mr. Soros, Mr. Neier said.

Write to Michael M. Phillips at michael.phillips@wsj.com2

URL for this article:

http://online.wsj.com/article/0,,SB110955216307465482,00.html

Hyperlinks in this Article:

(1) http://online.wsj.com/article/0,,SB110955131006765463,00.html

(2) mailto:michael.phillips@wsj.com

(3) http://online.wsj.com/article/0,,SB110955131006765463,00.html

Copyright 2005 Dow Jones & Company, Inc. All Rights Reserved

This copy is for your personal, non-commercial use only. Distribution and use of this material are governed by our Subscriber Agreement and by copyright law. For non-personal use or to order multiple copies, please contact Dow Jones Reprints at 1-800-843-0008 or visit www.djreprints.com.

Subject: Washington Post Weighs In On Needle Exchange

Gentlefolk-- Below find a Washington Post editorial

about needle exchange, Souder hearings, and what the

writer calls the U.S. government's "bullying

flat-earthism." It appears in the Sunday paper, so

those "opinion leaders' who missed Saturday's New York

Times should see this one. While it doesn't mention

the CND as explicitly as the Times, it sets the stage

well.

Best,

Daniel

Deadly Ignorance

Sunday, February 27, 2005; Page B06

THE BUSH administration is quietly extending a policy

that undermines the global battle against AIDS. It is

being pushed in this direction by Congress, notably by

Rep. Mark Edward Souder (R-Ind.). But some

administration officials zealously defend this policy

error, claiming scientific evidence that doesn't

exist.

The administration's error is to oppose the

distribution of uncontaminated needles to drug

addicts. A large body of scientific evidence suggests

that the free provision of clean needles curbs the

spread of AIDS among drug users without increasing

rates of addiction. Given that addicts are at the

center of many of the AIDS epidemics in Eastern Europe

and Asia, ignoring this science could cost millions of

lives. In Russia, as of 2004, 80 percent of all HIV

cases involved drug injectors, and many of these

infections occurred because addicts share contaminated

needles. In Malaysia, China, Vietnam and Ukraine, drug

injectors also account for more than half of all HIV

cases. Once a critical mass of drug users carries the

virus, the epidemic spreads via unprotected sex to

non-drug users.

The administration claims that the evidence for the

effectiveness of needle exchange is shaky. An official

who requested anonymity directed us to a number of

researchers who have allegedly cast doubt on the

pro-exchange consensus. One of them is Steffanie A.

Strathdee of the University of California at San

Diego; when we contacted her, she responded that her

research "supports the expansion of needle exchange

programs, not the opposite." Another researcher cited

by the administration is Martin T. Schechter of the

University of British Columbia; he wrote us that "Our

research here in Vancouver has been repeatedly used to

cast doubt on needle exchange programs. I believe this

is a clear misinterpretation of the facts." Yet a

third researcher cited by the administration is Julie

Bruneau at the University of Montreal; she told us

that "in the vast majority of cases needle exchange

programs drive HIV incidence lower." We asked Dr.

Bruneau whether she favored needle exchanges in

countries such as Russia or Thailand. "Yes, sure," she

responded.

The Bush administration attempted to bolster its case

by providing us with three scientific articles. One,

which has yet to be published in a peer-reviewed

journal, was produced by an author unknown to leading

experts in this field who is affiliated with a group

called the Children's AIDS Fund. This group is more

renowned for its ties to the Bush administration than

for its public health rigor: As the Post's David Brown

has reported, it recently received an administration

grant despite the fact that an expert panel had deemed

its application "not suitable for funding." The two

other articles supplied by the administration had been

published in the American Journal of Public Health.

Although each raised questions about the certainty

with which needle-exchange advocates state their case,

neither opposed such programs.

Evidence that the administration does not cite leaves

little doubt about the case for needle exchange. A

study of 81 cities published in 1997 in the Lancet, a

medical journal, found that in cities without

needle-exchange programs, HIV infection rates among

injection drug users rose by nearly 6 percent per

year; by contrast, cities that had introduced

free-needle programs witnessed a decrease in infection

rates of about the same magnitude. Elias A. Zerhouni,

the director of the National Institutes of Health,

wrote last year that exchange programs "can be an

effective component of a comprehensive community-based

HIV prevention effort," and a World Health

Organization technical paper agreed that the provision

of clean needles and syringes should be "a fundamental

component of any comprehensive and effective

HIV-prevention programme." Addressing legitimate

methodological questions about the research favoring

needle exchange, the WHO reasonably concluded that

incomplete scientific evidence does not confer the

freedom to ignore the knowledge we do have.

Respecting science does not appear to be the

administration's priority, however. Not only is it

refusing to spend federal dollars on needle exchange,

but the administration also is waging a campaign to

persuade the United Nations to toe its misguided line.

The U.N. Office on Drugs and Crime, which is heavily

reliant on U.S. funding, has been made to expunge

references to needle exchange from its literature, and

the administration is expected to continue its

pressure on the United Nations at a meeting that

starts March 7. The State Department's new leadership

needs to end this bullying flat-earthism. It won't

help President Bush's current effort to relaunch his

image among allies. And it's almost certain to kill

people

Editorial

The New York Times

February 26, 2005

http://www.nytimes.com/2005/02/26/opinion/26sat1.html?th

 The Bush administration has contributed to suffering and death through the so-called global gag rule, which prohibits Washington from giving money to any group that performs - or even talks about - abortions. Organizations that provide desperately needed family planning and women's health services have lost their financing. Now there are moves in Congress and inside the administration to apply a similar rule to needle exchange programs. That would be an even more deadly mistake.

Allowing drug users to trade used needles for clean ones gets dangerous needles off the street and minimizes needle sharing. A proven weapon against AIDS transmission, it has not been shown to increase drug use, and indeed may reduce drug addiction by providing a way to talk to drug users and lead them to treatment. It is endorsed by virtually every mainstream public health group.

Getting users into drug treatment is the best way to keep them safe. But the push for treatment - which is expensive and difficult - should come with needle exchanges.

Drug use is not a significant source of AIDS infection in Africa. In parts of Asia, the former Soviet bloc and Eastern Europe, needles are the major source of infection; three-quarters of all newly infected people in Russia

are intravenous drug abusers, as are half of those newly infected in China. These are just the places where the AIDS epidemic is likely to explode next. A bumper poppy crop in Afghanistan will worsen the outlook, producing cheap heroin that could turn opium smokers into heroin injectors and thus fuel the epidemic.

Opponents of needle exchanges, mainly among the religious right, argue that the practice muddies the message that illegal drug use is unacceptable, and keeps drug abusers from suffering the consequences of their addiction. By this twisted logic, doctors should refuse to treat lung cancer in smokers. In any case, AIDS infections from sharing needles are not limited to drug users. They infect sexual partners, spreading the epidemic through

societies.

While Washington does not buy syringes for needle-exchange programs, it does give money to groups that use other people's money to administer needle exchanges. But some conservatives are attempting to stop even that. The assistant secretary of state for international narcotics and law enforcement, Robert Charles, warned the United Nations Office on Drugs and Crime, which currently holds the rotating chairmanship of the joint program

Unaids, that the organization should not work on needle exchange issues and should remove positive references to them from its Web site, which it did.

Representatives Mark Souder of Indiana and Tom Davis of Virginia, both Republicans, have asked the United States Agency for International Development for details on all financing for programs in which any group strongly advocating needle exchanges also participates.  These lawmakers claim that a U.N. drug agency report attacks needle exchange as encouraging drug use. In fact, the report makes no such accusation and endorses needle exchanges.

In the Senate, a member of the staff of Sam Brownback, the Kansas Republican, has compiled a grossly inaccurate chart of programs financed by the Global Fund to fight AIDS, Tuberculosis and Malaria that is subtitled "Immoral, Illegal (with bilateral funds) or Inconsistent with U.S. Foreign Policy." Needle exchanges rank high. At the moment, Mr. Brownback's office says he does not intend to attempt to block these programs. But some newer right-wing lawmakers are considering it.

So far, attempts to eliminate needle-exchange programs overseas seem to have limited support. Many administration officials and conservatives in Congress do not want to see crucial AIDS prevention measures

derailed or American support withdrawn from such organizations as the Global Fund. One important test will be what the administration does in early March at the annual meeting of the United Nations Commission on Narcotic Drugs. Last year, United States representatives there attacked the scientific evidence in favor of needle exchanges as unconvincing. This year, the United States should refrain from such attacks - and members of

Congress should call off their budding witch hunt.

Washington's antipathy toward needle exchanges is a triumph of ideology over science, logic and compassion. The United States should help pay for these important programs. If it cannot bring itself to do so, it should at least allow the rest of the world to get on with saving millions of lives.

February 21, 2005

Mr. Akira Fujino

Regional Representative for East Asia and the Pacific

United Nations Office on Drugs and Crime

United Nations Building, 3rd Floor

Rajadamnern Nok Avenue

Bangkok 10200, Thailand

*Sent via fax: (02) 281 2129*

Dear Mr. Fujino,

We are writing to express our deep concern about an apparent shift in the United Nations Office on Drugs and Crime (UNODC)’s priorities related to the prevention of HIV/AIDS among drug users. As you know, the weeks leading up to the 2005 Commission on Narcotic Drugs have seen widespread alarm among HIV/AIDS experts and service providers about U.S. pressure on UNODC to curb its support for proven HIV prevention strategies for injection drug users. We are writing to elaborate our particular concerns about UNODC’s apparent suspension of a regional project and task force in East Asia and the Pacific on drug use and HIV vulnerability.

The region of East Asia and the Pacific is home to an estimated 2 million to 4 million injection drug users, of whom some 750,000 are living with HIV/AIDS. This figure is projected to double by 2010 absent aggressive strategies to reduce syringe sharing among injection drug users and promote comprehensive harm reduction and risk reduction strategies. Currently, however, an estimated 1 percent of injection drug users in the region currently have access to these services. Injection drug use now accounts for the overwhelming majority of new HIV infections in most Asian countries, from 59 percent in Vietnam to 64 percent in China to 76 percent in Malaysia. In numerous countries in the region, HIV prevalence among injection drug users is well over 50 percent.

HIV vulnerability among injection drug users is everywhere fueled by abuses of due process and other human rights. In Thailand in 2003, a brutal “war on drugs” not only left close to 3000 suspected drug offenders shot dead in apparent extrajudicial executions, but also drove countless drug users into hiding where they could not obtain HIV prevention services. In China, drug users are routinely tested for HIV without their consent or knowledge in forced detoxification centers. In Vietnam, a state-sponsored campaign against “social evils” authorizes police to detain suspected drug users and commit them to forced rehabilitation centers for at least five years. Such actions not only violate human rights in themselves, but also impede drug users’ ability to protect themselves from HIV/AIDS and other blood-borne diseases.

Given the daily interface between injection drug users and the criminal justice system, the law enforcement community plays a critical role in reducing drug users’ HIV risk. Practices such as planting drugs on suspected drug users, forcing drug users to sign false confessions, and extorting money from criminal suspects can force drug users into hiding and impede their access to HIV prevention services. Confiscating sterile syringes from injection drug users, a practice that appears to be widespread in the region, can lead to increased syringe reuse and sharing and elevated HIV risk. Detaining drug users for long periods of time can lead to widespread syringe sharing among detainees, particularly if accurate information and HIV prevention services are not provided in jails, prisons, or forced treatment centers.

As chair of the Joint United Nations Programme on HIV/AIDS (UNAIDS) Committee of Co-sponsoring Organizations and the lead United Nations agency providing technical assistance to national governments on narcotics enforcement, UNODC is the natural entry point for any dialogue between law enforcement officials and HIV/AIDS service providers. Through the regional project on “Reducing HIV Vulnerability from Injection Drug Use,” successfully coordinated by Mr. Wayne Bazant, UNODC sought to promote this dialogue by developing regional drug demand reduction plans, advocating for partnerships between health and police officials, highlighting the public health dimension of drug criminalization laws, providing recommendations to governments and the United Nations Office of the High Commissioner for Human Rights on rights-based approaches to injection drug use, and contributing to the development of national-level multi-sectoral working groups on drug use and HIV/AIDS. Mr. Bazant, in particular, was widely admired by governments, United Nations personnel, and local HIV/AIDS and drug-user organizations for involving a wide range of stakeholders in UNODC’s demand reduction projects and for his willingness to serve as a resource on all aspects of injection drug use and HIV risk.

UNODC’s decision to discontinue Mr. Bazant’s contract as well as the project that he has very successfully led represents a significant setback for HIV prevention at a critical time in Asia’s AIDS epidemic. Given UNODC’s leadership role in UNAIDS and the emphasis on HIV prevention at the upcoming meeting of the Commission on Narcotic Drugs, the elimination of a project “Reducing HIV Vulnerability from Drug Abuse” in Southeast Asia raises serious questions about how UNODC is meeting its mandate to reduce HIV risk among injection drug users. The decision not to renew the contract of Mr. Bazant comes at a time of renewed community concern about UNODC’s commitment to the full range of drug demand reduction and HIV prevention strategies. We urge you to provide clear and public details on how you propose to meet the mandate your agency's mandate for HIV prevention among drug users in Southeast Asia and more generally.

Yours truly,

Thai Drug Users’ Network (TDN)

Thai AIDS Treatment Action Group (TTAG)

cc. Antonio Maria Costa, UNODC Executive Director

Christian Kroll, Head of UNODC HIV/AIDS Unit

Peter Piot, UNAIDS Executive Director

--

INVITATION

to the

# Vienna NGO Committee on Narcotic Drugs Forum

Wednesday, 9th March 2005, Conference Room I, Vienna International Centre

## AGENDA

# “To reduce the harmful consequences of drug misuse”

The meeting will be chaired by Eric Carlin, The Mentor Foundation.

10.00 Welcome and introductions Eric Carlin (UK) &

Eva Tongue (Chairperson)

10.15 Representative of UNODC

10.45 HIV prevention and drug abuse – experience from Lebanon Elie Araj (Lebanon)

11.15 Community participation in developing drugs strategies David Turner (Italy)

11.45 Practical impact on the reduction of drug-related harm Gabriele Gottwald Nathaniel (Austria)

12.15 Discussion

13.00 Lunch and opportunity to network

15.00 Education, prevention and harm reduction Adrian King (UK)

15.30 A national approach – Hungarian experience Zsolt Demetrovich

(Hungary)

16.00 Reduction of drug-related harm - the philosophical Christian Haring

aspect (Austria)

16.30 International anti-trafficking efforts – experience from Alexander)

Kyrgyzstan and Central Asia Zelitchenko

(Kyrgyzstan)

17.00 Discussion

17.30 Summing up and recommendations Eric Carlin (UK)

17.45 Close

Volume 365, Issue 9460 , 19 February 2005, Pages 629-630

Losing tolerance with zero tolerance

The Lancet

Available online 19 February 2005.

### In 1998, the UN General Assembly pledged the "elimination or significant

reduction" of illegal drug production and use within 10 years. With just 3

years until this deadline, around 200 million people still take illegal

drugs each year. Huge resources have been poured into trying to stem the

supply of illegal drugs through drug-traffic interdiction, the prosecution

of dealers, and the imprisonment of hundreds of thousands of users-yet the

supply and abuse of illegal drugs has escalated.

The dominant role played by influential nations that still favour zero

tolerance, such as the USA and China, means it is difficult to change

international drug-control policies. The USA is currently pressing the

International Commission on Narcotic Drugs to abandon its support for

needle-exchange programmes and harm-reduction strategies to prevent HIV.

Moreover, the strategies used by governments to curb the drug trade are

causing substantial health and social problems among those that depend on

illegal drug production to survive.

The framework for this failing global drugs policy was set by three UN

conventions in 1961, 1971, and 1988. Under the conventions, countries are

obliged to pursue growers, dealers, and users in an attempt to create a

"drug free world". Since 1995, various action plans implemented by the

European Union have also failed to counter the global increase in drug use

and related problems. Prohibition policies have torn apart and destabilised

drug-producing countries, and zero-tolerance approaches to drug use have

often created more harm to the user than the drug use they were intended to

prevent.

A new set of guidelines for the European-wide drugs strategy over the next 8

years is due to be published next month. But these guidelines repeat many of

the mistakes of previous drug-control efforts by putting law enforcement

before health.

Despite a frequent focus on the criminal aspects of drug addiction, the

public-health problems of illegal drug use are widespread. An estimated 4

million people depend on income derived from the cultivation of illegal drug

crops. In many countries, efforts to wipe out drug cultivation and supply

are at a high cost to human welfare and lives.

In Colombia, a decade of intense US-led efforts to eradicate coca production

through financial and military support to Colombia's Government has had no

real effect on overall cocaine production but a detrimental effect on human

health. Alongside a civil war that has killed over 51000 people in the past

2 years-to which the drug trade has contributed-herbicide aerial-spraying

campaigns have destroyed subsistence food crops along with coca plants and

commercial crops. The consequent loss of income and food has led to a

dramatic increase in malnutrition and other illnesses-as illustrated in a

recent study by Oxfam in North Santander's rural communities, which found

that 87% of children under 7 years were either malnourished or below normal

weight.

Afghanistan is also struggling to wean farmers off opium production. 2004

saw a rise in Afghanistan's opium production for the third year running

according to the UN Office on Drugs and Crime (UNODC). Despite money being

thrown at poppy farmers to grow alternative crops, opium is viewed as low

risk-its price never normally falls below a certain level, it needs little

water, and it is easy to store-and there are no alternative crops that

generate equivalent income.

An escalating problem in those developing countries that produce the illegal

drugs is the increasing number of domestic addicts. In 2003, the UNODC's

lowest estimate for drug users in Kabul alone was 63000. The problem is

worsened by the lack of preventive or treatment services.

A report by the International Narcotics Control Board in 2003, urged richer

countries to give Afghanistan more aid to help it wipe out poppy growing.

According to Habibullah Qadiri, head of Afghanistan's counter-narcotics

ministry, an $870 million programme announced by the USA is little more than

a rumour. In fact the US Government has talked about providing $800 million,

and most of this is for spraying poppy fields. The funding has not yet been

approved by Congress.

For farmers there are few economic incentives for growing alternatives to

drug crops; for example, the price of alternative legal crops such as coffee

often slumps below the cost of production. This situation is exacerbated by

agricultural trade rules, policies, and practices that make it impossible to

earn a living from legal crops in many developing countries. Under current

trading arrangements, farmers in most developing countries are faced with

falling crop prices, because subsidised goods from Europe and the USA are

flooding the market, and tariffs effectively tax goods imported from poor

countries at four times the rate of goods produced in rich countries.

Nations that preach free trade and access to markets for drugs such as

alcohol and tobacco are among those that advocate punitive trade and

diplomatic policies towards the poor countries that provide illegal drugs.

The UNODC has attempted to tackle these issues by introducing the

Alternative Development Programme. This aims to improve the "socioeconomic

quality of life of targeted populations through integrated development

projects"-programmes that encourage growers to switch to alternative legal

profitable crops that provide a licit source of income. These programmes

take a holistic approach that includes safe water, health, education, and

rural infrastructure. But a lack of donor interest in funding legal

livelihood initiatives-despite small and successful projects in southeast

Asia and Latin America-is a major hurdle to widespread success, according to

the UNODC, and drug cultivation remains a flourishing industry.

Drug abuse is a public-health problem in the countries that supply as well

as those that consume the drugs. There must be a collective responsibility

to ensure that the citizens of developing nations do not pay the price for a

high demand for drugs in developed countries through drug-control policies

that destroy livelihoods and their health, while having little impact on

drug use. Individual countries and development agencies must invest in

making drug control part of a broader humanitarian effort that supports

alternative development programmes and makes poverty reduction a serious

aim.
