# Supplementary material for: Witch-hunt
Source: Harm Reduct J. 2005 Mar 4;2:3. doi: 10.1186/1477-7517-2-3 (PMC555579; doi:10.1186/1477-7517-2-3)
Supplement: Additional File 2 — French version of the Open Letter [file 1477-7517-2-3-S2.pdf]

le 1er mars 2005

Lettre ouverte aux Délégués de la dix-huitième session de la Commision sur les Drogues Narcotiques (CND)

En cette année où l'UNODC (Office des Nations Unies contre la drogue et le crime) préside l'exécutif du programme joint des Nations Unies sur le VIH/Sida (ONUSIDA), nous tenons à manifester notre inquiétude quant aux efforts que font les Etats-Unis afin de contraindre l'UNODC à retirer son soutien aux échanges de seringues, à la fourniture de méthadone, ou à d'autres mesures ayant prouvé leur efficacité pour contenir la diffusion du VIH chez les usagers de drogue. L'usage de drogue par injection s'avère être la cause de la majorité des contaminations par le VIH dans des douzaines de pays en Asie, ou dans l'Ex Union Soviétique, dont la Russie, la Chine, tous les pays d'Asie centrale, et la plupart de l'Asie du Sud-Est. Dans la plupart des pays hors Afrique, les nouvelles contaminations concernent aujourd'hui principalement les usagers de drogues par voie intraveineuse. Comme le directeur de l'UNODC Antonio Maria Costa le faisait remarquer lors de la Conférence internationale de Juillet 2004, une réponse efficace à l'épidémie de Sida due aux injections requiert une prévention accrue, incluant l'échange de seringues, plutôt que des politiques qui ne font qu'accélérer la diffusion du VIH par des emprisonnement massifs et aveugles.

Malheureusement, les événements récents suggèrent que l'UNODC –sous la pression des Etats-Unis- soit appelé à retirer son soutien à des stratégies préventives ayant fait leurs preuves, précisément au moment où le besoin d'un engagement accru en échange de seringues et en traitement de substitution aux opiacées se fait sentir. Il est particulièrement préoccupant que l'UNODC soit silencieuse l'année où l'agence préside le Comité des organisations sponsors de l'ONUSIDA, et l'année où le débat thématique des 48èmes rencontres du CND doivt mettre l'accent sur la prévention du VIH. Parmi d'autres éléments qui ont grandement provoqué notre inquiétude, se trouvent :

- M. Costa, qui avait l'année passée exprimé son soutien à des changements positifs du code pénal russe, à l'extension de l'échange de seringues dans les pays confrontés à des épidémies dues aux injections, et à d'autres mesures de réduction des risques liés à la drogue, a visiblement été influencé par le Département d'Etat américain. Suite à une rencontre avec Robert Charles, le Secrétaire adjoint aux Affaires relatives aux narcotiques internationaux et à l'exécution de la Loi, M. Costa se mit à plaider pour une révision de tous les documents UNODC, électroniques ou imprimés, faisant référence à la « *réduction des risques* », et prit le parti d'être « *encore plus vigilant à l'avenir* ».
- En Asie du Sud-Est, UNODC a suspendu un programme qui visait à réduire la vulnérabilité au VIH des usagers de drogue à travers des approches qui privilégiaient la santé publique et les Droits de l'Homme pour les usagers de drogue, plutôt q'une politique de sanction.
- Même l'échange de seringues, affiché par l'ONUSIDA, l'OMS, et les membres des Nations Unies, comme une partie essentielle et efficace de la prévention du VIH, est devenu « politiquement incorrect ». Un e-mail émanant d'un senior de l'UNODC et destiné à un junior, demandait à ce dernier de

> *« s'assurer que les références à la réduction des risques, et à l'échange de seringues sont bien évitées dans les documents, publications et positions de l'UNODC ».*

Nous avons conscience que l'UNODC est dépendante des contributions des nations donatrices, et que les Etats-Unis sont les plus grands donateurs pour le contrôle des drogues des Nations Unies. En même temps, la vie de centaines de milliers de personnes dépend d'une approche raisonnée et scientifique de la prévention du VIH. De nombreuses études, dont certaines émanant du gouvernement américain lui-même, ont montré que les stratégies telles que l'échange de seringues et la distribution de méthadone diminuaient manifestement la transmission du VIH et les autres risques sanitaires. Le simple fait que les délégués américains déclarent ces preuves en faveur de l'échange de seringues « *non convaincantes* », ainsi qu'ils le firent l'année dernière lors d'une session du CND, ne devraient pas permettre de déterminer la politique de contrôle des drogues de l'ONU, et les efforts de prévention du VIH, qui sont inextricablement et intrinsèquement liées. De même, l'UNODC ,un co-sponsor de l'ONUSIDA, et, qui plus est, une agence avec un rôle essentiel à jouer sur le front de la lutte contre le VIH, ne devrait pas davantage se voir demander de revenir sur ses positions publiques quant à l'échange de seringues, pour la seule raison que celles-ci ne conviennent pas à ce que les Etats-Unis estiment acceptable.

Les stratégies qui se contentent de viser à l'arrêt de la consommation de drogue ne constituent pas une alternative acceptable aux programmes tels que l'échange de seringues, qui aident, eux, les consommateurs actifs à se protéger du VIH/Sida. L'expérience prouve que la « tolérance zéro » en matière de contrôle des drogues peut surtout avoir pour effet de conduire les consommateurs à la clandestinité, de les éloigner des traitements anti-drogue et des autres services de santé. Ceci est particulièrement vrai, quand, comme c'est le cas dans beaucoup de pays, les mesures anti-drogue entraînent des arrestations injustifiées, des actes de violence et des extorsions de la part de la police, des détentions prolongées sans jugement, des traitements anti-drogue contraints, des incarcérations disproportionnées dans de cruelles conditions, et dans certains cas, des exécutions extra-judiciaires. Les programmes comme l'échange de seringues et les substituts aux opiacés, à l'inverse, permettent à la fois de prévenir la transmission du VIH, et de bâtir une passerelle avec les autres services de santé. Restreindre ces programmes correspond à une violation criante du Droit de l'Homme à la Santé pour les consommateurs de drogue.

Lorsque vous vous rassemblerez cette année pour débattre de prévention du VIH/Sida et de l'abus de drogue, nous vous prions de bien vouloir soutenir l'échange de seringues, les produits de substitution, et les autres méthodes de réduction des risques ayant prouvé leur efficacité dans la lutte contre le VIH ; d'affirmer le droit humain des usagers de drogue à la Santé et aux services de soins ; et de rejeter les efforts visant à supplanter la science, et à lier les mains de ceux qui travaillent sur le front de la lutte contre le VIH. Rien de moins que l'avenir de l'épidémie de VIH est ici en jeu.

cc: Programme Joint de l'ONU contre le HIV/AIDS
Organisation Mondiale de la Santé
Bureau du Haut Commissaire pour les Droits de l'Homme
*International Narcotics Control Board

Les organisations et individus suivants ont signé cette lettre avant le 1er mars 2005:

Organisations par région :

Asie

AIZHIXING Institute of Health Education, Beijing, China

Asian Harm Reduction Network (AHRN), Chiang Mai, Thailand

Asia Pacific Rainbow, New Delhi, India

Australian Drug Foundation, Melbourne, Australia

Australian Injecting and Illicit Drug Users League, Darlinghurst, Australia

Blue Diamond Society, Kathmandu, Nepal

Burnet Institute, Melbourne, Australia and Yangon, Myanmar

The Centre for Harm Reduction, Macfarlane Burnet Institute for Medical Research & Public Health, Melbourne, Australia

Community Health, Rehabilitation, Education & Awareness (CREA), Dhaka, Bangladesh

Drug Action Committee of the City of Greater Geelong, Victoria, Australia

Family Drug Support, Willoughby, Australia

Health and Development Networks (HDN), Chiang Mai, Thailand

ILGLAW – Asia, New Delhi, India

Lawyers Collective HIV/AIDS Unit, New Delhi, India

Malaysian AIDS Council, Kuala Lumpur, Malaysia

Malaysian Harm Reduction Working Group, Kuala Lumpur, Malaysia

Northern Sydney Central Coast Health, Sydney, Australia

Pinoy Plus Association, Manila, Philippines

RISE, Peshawar, Pakistan

Thai AIDS Treatment Action Group (TTAG), Bangkok, Thailand

Thai Drug Users' Network (TDN), Bangkok, Thailand

Turning Point Alcohol and Drug Centre, Melbourne, Australia

VIVAIDS Inc., Fitzroy, Australia

WartaAIDS, Jakarta, Indonesia

Western Australian Substance Users Association, Bunbury, Australia

Yayasan Spiritia, Jakarta, Indonesia

Afrique

AfriCASO: African Council of Aids Service Organization, Dakar, Senegal

Kenya AIDS NGOs Consortium (KANCO), Nairobi, Kenya

Sheryl's Orphans Children Home, Nairobi, Kenya

UNDP/Kinshasa, Kinshasa, Democratic Republic of Congo

Europe/Asie Centrale

Abraço—Associação de Apoio a Pessoas Infectadas e Afectadas pelo VIH /SIDA, Lisbon, Portugal

Act Up—Paris, Paris, France

Action Against AIDS Germany, Tubingen, Germany

Actions Traitements, Paris, France

AGIHAS (PLWHA Support group), Riga, Latvia

AIDES NGO, Pantin Cedex, France

AIDS Action Europe, Amsterdam, Netherlands

AIDS Foundation East-West (AFEW), Moscow, Russia

Aids-Hilfe Bonn e. V., Bonn, Germany

AKZEPT E.V.—Bundesverband für akzeptierende Drogenarbeit und humane Drogenpolitik, Berlin, Germany

Amsterdam Institute for Addiction Research (AIAR), Amsterdam, Netherlands

A.N.O., Prague, Czech Republic

Associazione Mastropietro & Co., Turin, Italy

Associazione Nazionale Italiana Lotta AIDS (A.L.A.), Milano, Italy

Blupoint Drug Counselling Centre, Budapest, Hungary

Bremen Institute for Drug Research, Bremen, Germany

CA Odyseus, Bratislava, Slovakia

Center for Interdisciplinary Research on Women and Gender (ZFG), Oldenburg, Germany

Central and Eastern European Harm Reduction Network, Vilnius, Lithuania

The Centre for Research on Drugs and Health Behaviour, Imperial College, United Kingdom

Charitable Foundation "Rehabilitation Center of Drug Addicts "Virtus", Dnipropetrovsk, Ukraine

The Chrysalis Drug Project, Hertford, United Kingdom

Coalition on Vulnerable Population "I Can Live", Vilnius, Lithuania

Community Organization of People Living with HIV & AIDS, Moscow, Russia

Convictus Eesti, Tallinn, Estonia

Cranstoun Drug Services, London, United Kingdom

DIA+LOGS NGO, Support centre for those affected by HIV/AIDS, Riga, Latvia

Die Deutsche Gesellschaft für Suchtmedizin (vorm DGDS) e.V., Hamburg, Germany

DroBeL - Drogenberatung Lehrte e. V., Lehrte, Germany

Drogenberatung e.V., Bielefeld, Germany

Drogprevenciós Alapítvány, Budapest, Hungary

Dublin AIDS Alliance Ltd., Dublin, Ireland

Equal to Equal, Almaty, Kazakhstan

Estima Associação, Leiria, Portugal

Északi Támpont Egyesület, Budapest, Hungary

European AIDS Treatment Group, Brussels, Belgium

European Coalition for Just and Effective Drug Policies (ENCOD), Antwerp, Belgium

European Commission, Coordination of Anti-drugs Policy, Directorate-General Justice, Freedom and Security, Brussels, Belgium

Evangelisches Stadtjugendpfarramt Gera, Gera, Germany

Förderverein interdisziplinärer Sucht- und Drogen-forschung (FISD) e.V., Zentrum für interdisziplinäre Sucht-und drogen-forschung – ZIS, Hamburg, Germany

Freundes- und Förderkreis Suchtkrankenhilfe e.V., Wuppertal, Germany

GAT - Grupo Português de Activistas sobre Tratamentos de VIH/SIDA, Lisbon, Portugal

George House Trust, Manchester, United Kingdom

Grazia Zuffa, Fuoriluogo/Il Manifesto, Rome, Italy

Greater Glasgow Drug Action Team, Glasgow, Scotland

Grup Igia, Barcelona, Spain

Grupo de Trabajo sobre Tratamientos del VIH (gTt), Barcelona, Spain

Health and Social Development Foundation, Sofia, Bulgaria

Health Education Association NGO, Yerevan, Armenia

Helsinki Foundation for Human Rights, Warsaw, Poland

Hemp Seed Association, Budapest, Hungary

HIV Prevention Programme at Kekava, Riga, Latvia

Hope-Sofia, Sofia, Bulgaria

Indro e.V., Münster, Germany

Initiative for Health Foundation, Sofia, Bulgaria

The Initiative of Drug Users' Mutual Support, Vilnius, Lithuania

The Institute for Criminal Policy Research (ICPR), School of Law, King's College, London, United Kingdom

Institut für Sozialpädagogik, Technische Universität Berlin, Berlin, Germany

The International Community of Women Living with HIV/AIDS (ICW), London, United Kingdom

International Harm Reduction Association, London, United Kingdom

Irish Penal Reform Trust, Dublin, Ireland

Itaca Europe, Rome, Italy

Italian League for the fight against AIDS - Center for Human Rights and Public Health (LILA CEDIUS ONLUS), Milan, Italy

JES e.V., Bielefeld and Bremen, Germany

John Mordaunt Trust, London, United Kingdom

Mainline Foundation, Amsterdam, Netherlands

Malopolskie Stowarzyszenie PROBACJA (PROBACJA Association), Krakow, Poland

MARATON, Warsaw, Poland

Methadone Alliance, London, United Kingdom

MODUS VIVENDI, Prévention du sida et réduction des risques pour usagers de drogues, Brussels, Belgium

Monar Krakow Drugs Project, Krakow, Poland

MONAR, Pulawy, Poland

National AIDS Trust, London, United Kingdom

Netherlands Drug Policy Foundation (SDB), Haarlem, Netherlands

NGO TRUST, Skopje, Macedonia

Osservatorio Italiano sull'Azione Globale contro l'AIDS, Rome, Italy

PASSAGE, Skopje, Macedonia

Plymouth Drug and Alcohol Action Team, Plymouth, United Kingdom

Quest for Quality, Amsterdam, Netherlands

REFORM, Drug Policy Interest Group, Essex, United Kingdom

RFSU, The Swedish Association for Sexuality Education, Stockholm, Sweden

Romanian Association against AIDS (ARAS), Bucharest, Romania

Romanian Harm Reduction Network (RHRN), Bucharest, Romania

Russian Harm Reduction Network, Moscow, Russia

SANANIM, Prague, Czech Republic

Sensoa (Flemish Centre for Expertise and Services on Sexual Health and HIV), Antwerp, Belgium

SIDACTION – International Programs, Paris, France

SIDA STUDI, Barcelona, Spain

SignPost Forth Valley, Stirling, Scotland

Social AIDS Committee, Warsaw, Poland

SOMA – Associação Portuguesa Anti-proibicionista, Lisbon, Portugal

Steunpunt Druggebruikers, Antwerp, Belgium

T3E [UK] Ltd., London, United Kingdom

Transform Drug Policy Foundation, Bristol, United Kingdom

Transnational Institute (TNI) Drug and Democracy Programme, Amsterdam, Netherlands

Tver AIDS Center, Tver, Russia

Tver Center of Drugs Addiction Treatment, Tver, Russia

United Kingdom Harm Reduction Alliance Eccles, Kent, United Kingdom

Women for Women's Human Rights (WWHR) - New Ways, Istanbul, Turkey

World AIDS Campaign, Amsterdam, The Netherlands

Zentrum für interdisziplinäre Frauen- und Geschlechterforschung – ZFG, Carl von Ossietzky Universität Oldenburg, Oldenburg, Germany

Zentrum Suchtmedizin Klinikum Wahrendorff, Sehnde, Germany

Les Amériques

Argentinean Harm Reduction Association (ARDA), Buenos Aires, Argentina

Argentinean Harm Reduction Network (REDARD), Buenos Aires, Argentina

Asociación Civil “Convivencia” de Personas Viviendo y Conviviendo con VIH/SIDA, Buenos Aires, Argentina

Brazilian Drug Users Network, Recife, Brazil

Fundación para Estudio e Investigación de la Mujer. Buenos Aires, Argentina

HUMANAR, Huritaba, Brazil

Intercambios Asociación Civil, Buenos Aires, Argentina

International Women Aids Caucus, Buenos Aires, Argentina

LACCASO - Latin American and Caribbean Council of AIDS Service Organizations, Caracas, Venezuela

Mujer y Salud en Uruguay (MYSU), Montevideo, Uruguay

ONG INTERPARES, Parana Entre Rios, Argentina

Parana Drug Users Network, Curitiba, Brazil

Programa Alter-Acciones, El Abrojo – Instituto de Educación Popular, Montevideo, Uruguay

REDUC, Brazilian Harm Reduction Network, São Paulo, Brazil

RELARD, Latin American Harm Reduction Network, Curitiba, Brazil

Moyen-Orient

Iranian National Center for Addiction Studies (INCAS), Tehran, Iran

The Moroccan Association for the Fight Against AIDS, Marrakesh, Morocco

Persepolis Harm Reduction NGO, Tehran, Iran

Persia+, UNDP GIPA Programme, Tehran, Iran

Amérique du Nord

Access Works! Harm Reduction Services, Minneapolis, MN, USA

Act Up—East Bay, Oakland, CA, USA

Advocates for Recovery Through Medicine, Connecticut Chapter, New London, CT, USA

After Hours Project, Inc., Brooklyn, NY, USA

AIDS Foundation of Chicago, Chicago, IL, USA

AIDS Project Los Angeles, Los Angeles, CA, USA

AIDS Treatment Data Network, New York, NY, USA

American Academy of HIV Medicine, Washington DC, USA

American Foundation for AIDS Research, Washington DC, USA

ARC International, Ottawa, Ontario, Canada

Canadian Harm Reduction Network, Toronto, Ontario, Canada

Canadian HIV/AIDS Legal Network, Montreal, Quebec, Canada

Center for Health and Gender Equity (CHANGE), Takoma Park, MD, USA

Center for Human Rights and Public Health, Johns Hopkins Bloomberg School of Public Health, Baltimore, MD, USA

CHAMP (Community HIV/AIDS Mobilization Project), New York, NY, USA

Chicago Recovery Alliance, Chicago, IL, USA

CitiWide Harm Reduction, Bronx, NY, USA

Drug Overdose Prevention & Education Project, San Francisco, CA, USA

DrugSense, Irvine, CA, USA

Exponents, New York, NY, USA

Foundation for Integrative AIDS Research (FIAR), Brooklyn, NY, USA

Gay Men's Health Crisis, New York, NY, USA

Global AIDS Alliance, Washington DC, USA

Harm Reduction Coalition, New York, NY, USA

Harm Reduction Project, Denver and Salt Lake City, UT, USA

Health GAP (Global Access Project), New York, NY, USA

HIV Advocacy Council of Oregon and SW Washington, Portland, OR, USA

HIV Resource Center, Roseburg Risk Reduction, Roseburg, OR, USA

Housing Works, Inc., New York, NY, USA

Human Rights Watch, New York, NY, USA

International Antiprohibitionist League, New York, NY, USA and Rome, Italy

International Center for Advancement of Addiction Treatment, Baron Edmond de Rothschild Chemical Dependency Institute of Beth Israel Medical Center, New York, NY, USA

International Council of AIDS Service Organizations (ICASO), Toronto, Ontario, Canada

International Foundation for Alternative Research in AIDS (IFARA), Portland, OR, USA

International Gay and Lesbian Human Rights Commission, New York, NY, USA

International Women's Health Coalition, New York, NY, USA

Lower East Side Harm Reduction Center, New York, NY, USA

Montefiore Medical Center, Division of Public Health and Policy Research, Bronx, NY, USA

NAMA-Norcal, Santa Cruz, CA, USA

National Association of People with AIDS (NAPWA), Silver Spring, MD, USA

National Association for Victims of Transfusion-Acquired AIDS, Bethesda, MD, USA

The New York Academy of Medicine, New York, NY, USA

Open Society Institute, New York, NY, USA

Physicians for Human Rights, Washington, DC, USA

Positive Health Project, Inc., New York, NY, USA

Prevention Point Pittsburgh, Pittsburgh, PA, USA

TAG, Treatment Action Group, New York, NY, USA

Unified Networkers of Drug Users Nationally, Kingston, Ontario, Canada

Signataires Individuels

Vittorio Agnoletto, Scientific Mgr of LILA CEDIUS, Member of the European Parliament, GUE/NGL Group, Italy

Waheed Ahmad, Advocate High Court, Legal Secretary: World Asian Workers Organisation, Lahore, Pakistan

Joan Anderson, HIV/AIDS community Consultant and volunteer in Toronto, Ontario, Canada

Guillermo R. Aureano, Ph.D., Chercheur associé, Groupe d'étude et de recherche sur la sécurité internationale (GERSI), Département de science politique, Université de Montréal, Montréal, Quebec, Canada

Brambillaschi Barbara, L.I.L.A. - Lega Italiana per la Lotta contro l'AIDS-sede di Como, Italy

Trude Bennett, The University of North Carolina at Chapel Hill, School of Public Health, Chapel Hill, NC, USA

Dennis Berg, Ph.D., Professor of Sociology, California State University, Fullerton, CA, USA

Marie-Andrée Bertrand, Professor emeritus, University of Montreal, Criminology, Montréal, Quebec, Canada

Suzanne Bessoir, Harm Reduction Specialist, AIDS Service Center NYC, New York, NY, USA

Calum Blair, Harm Reduction worker, SignPost Forth Valley, Stirling, Scotland

Simon Boerboom, Psychiatrist, Amsterdam, Netherlands

Beth M. Bouloukos, Ithaca, New York, NY, USA

Scott Burris, James E. Beasley Professor of Law, Temple University Beasley School of Law, Philadelphia, PA, USA

Ryan Borgen, Student Global AIDS Campaign, Northeastern University School of Law, Boston, MD, USA

Jill Britton, London, United Kingdom

Damon Brogan, Manager, VIVAIDS, Melbourne, Australia

Benjamin Bynum, Fikelela AIDS Project, Cape Town, South Africa

Vincenzo Caracciolo, Associazione P24 Lila Livorno, Livorno, Italy

Craig Carmichael, The University of Queensland, Brisbane, Australia

Paul Causey, HIV/AIDS Program Consultant, Bangkok, Thailand

Jennifer Chapman, MPH, Project Manager/Research Coordinator, Center for Clinical, Epidemiology & Biostatistics, University of Pennsylvania, School of Medicine, Philadelphia, PA, USA

George K. Clarke, Volunteer CT Director, CMA, New London, CT, USA

Shelley Cogger, Research Assistant, Post Release Experience of Prisoners in Queensland (PREP-Q), Queensland Alcohol and Drug, Research and Education Centre (QADREC), University of Queensland, Australia

Franco Corleone, Forum Droghe, Rome, Italy

Melissa Dent, Community Development Worker, Melbourne, Australia

Tamara Desiatov, Koondoola, Western Australia

Kate Dickie, Annah Pickering, Managers, Auckland Branch of the New Zealand Prostitutes Collective, Auckland, New Zealand

Dr. Gyaw Htet Doe, Senior Consultant Psychiatrist, Taunggyi Drug Treatment Hospital, Taunggyi, Myanmar

Ms J Dowling, Auburn, NSW, Australia

Andy Dudley, Addaction, Derby, United Kingdom

Carolina Pecheny Durozier, Paris, France

Brian R. Edlin, M.D., Associate Professor of Medicine and Public Health, Center for the Study of Hepatitis C, Weill Medical College of Cornell University, New York, NY, USA

Maria Fotopoulou, Ph.D. student, Imperial College, University of London, London, United Kingdom

Mayada Youssef Fox, staff member, HIV/AIDS Department, WHO HQ, Geneva, Switzerland

Sandra Fox, former staff, Harm Reduction sector, Melbourne, Australia

Jonathan Freedlander, Baltimore, MD, USA

Liliana Gherman, MD, Program Director, Public Health Program, Soros Foundation, Chisinau, Moldova

Cees Goos, honorary consultant Anton Proksch Institut, former WHO staff, Vienna, Austria

Chris W. Green, Jakarta, Indonesia

Heath Greville (individual support), Department of Health, Perth, Western Australia

Patrick Griffiths, PhD candidate, Contemporary Globalisation and HIV/AIDS in Vietnam, RMIT University, Melbourne, Australia

Revd Dr Ian T Guy, General Practitioner, Fulcrum Medical Practice, Middlesbrough, United Kingdom

Bianca L. Guzman, Ph.D., CHOICES Director of Research, La Puente, CA, USA

Fiona Hale, International Network Manager, ICW, London, United Kingdom

Jen Hall, Information and Referral (Duty) Worker, Inner South Community Health Service, Prahran, Australia

Helena Hansen, MD-Ph.D. Candidate, Yale University School of Medicine, New Haven, CT, USA

Paul Hardacre, Training Officer, Asian Harm Reduction Network (AHRN), Chiang Mai, Thailand

Frank Harding, Ayer's Cliff, Quebec, Canada

Andy Hart, Vendafit Pty Ltd, Sydney, Australia

Paul J. von Hartmann, Project P.E.A.C.E., Planet Ecology Advancing Conscious Economics, Santa Rosa, CA, USA

Robert Heimer, Ph. D., Associate Professor, Department of Epidemiology and Public Health Center for Interdisciplinary Research on AIDS Yale University School of, New Haven, CT, USA

Beatriz Acevedo Holguin, Researcher on International Drug Policy, Hull, United Kingdom

Lital Hollander, Research Manager, ESMAN Medical Consulting, Milano, Italy

Danny Holness, Peer Education Officer, RaveSafe, Victoria, Australia

Daniel E. Hood, Ph.D., Department of Criminal Justice/Security Systems, State University of New York at Farmingdale, Farmingdale, New York, NY, USA

Annabelle Horton, UN Office of Drugs and Crime, Bangkok, Thailand.

Neil Hunt, Senior Research Associate, University of Kent; Honorary Research Fellow, Centre for Research on Drugs and Health Behaviour, Imperial College, London; Director, UK Harm Reduction Alliance Eccles, Kent, United Kingdom

Elena Jeffreys, Scarlet Alliance, Australias Sex Worker Association, Canberra Act, Australia

McKinzie McClay Jernberg, Minneapolis, MN, USA

Debbie Johnson, Macclesfield, Cheshire, United Kingdom

P. R. W. Kendall, MBBS, MSc, FRCPC, Provincial Health Officer, Ministry of Health Services, Victoria, British Columbia, Canada

Joseph Kim, Young Drug Users Peer Education Officer, VIVAIDS (Victorian Drug User
Organisation), Melbourne, Australia

Dr Stuart A. Kinner, Lecturer, Queensland Alcohol and Drug, Research and Education Centre (QADREC), Queensland, Australia

Debbie Kocziban, Addaction, Plymouth Devon, United Kingdom

Nicky Kupfer, Alcohol Tobacco and other drugs service, Sydney, Australia

Heather La Faye LPN, Medicine cures sickness,all the world is medicine, what is the self?, Recovery Resource Center, Minneapolis, MN, USA

Susana Lambrechts, Esteban Echeverría, Argentina

Deb Lapthorne, Director of Public Health, Plymouth Primary Care Trust/ Plymouth City Council, Plymouth, United Kingdom

Fiona Leibrick, Lecturer: Health Sciences (Alcohol & Other Drug Studies) / Registered Psychologist, Charles Darwin University, Darwin, Australia

Sarah Lippek, AIDS Center of Queens County, Long Island City, NY, USA

Deirdre Love, Senior Health Promotion Specialist HIV Positive People, Health First, London, United Kingdom

Wendy Loxley, Associate Professor, National Drug Research Institute, Curtin University of Technology, Bentley, West Australia

Devon MacFarlane, Vancouver, British Columbia, Canada

Catherine Mackenzie, St. Catharines, Ontario, Canada

Jan Chrostek Maj MD, Rydygier's Hospital, Krakow, Poland

Paolo La Marca, Project manager (Harm reduction & training), LILA CEDIUS (Italian league fight against AIDS), Milano, Italy

David Martin, Program Medical Officer, Health Canada, Vancouver, British Columbia, Canada

Phyra M. McCandless, Johns Hopkins School of Public Health, Baltimore, MD, USA

Rachel McLean, MPH Candidate, Johns Hopkins University Bloomberg School of Public Health, Baltimore, MD, USA

Belinda McNair, Senior Project Officer-City Drug Safety Plan, City of Melbourne, Melbourne, Australia

Cristina Menoyo, Secretaría del Plan Nacional sobre el Sida, Ministerio de Sanidad y Consumo, Madrid. España

Maria Luisa Milesi, Esteban Echeverría, Argentina

Dr Peter Miller, Senior Clinical Research Worker. National Addiction Centre (Maudsley Hospital/Institute of Psychiatry). King's College London, London, United Kingdom

PhDr. Michal Miovsky, Ph.D., Institute of psychology, Academy of Sciences of the Czech Republic, Prague, Czech Republic

Andrew Moss Ph.D, Professor of Epidemiology and Medicine, University of California, San Francisco, CA, USA

Marjorie "Mo" Mowlam, Kent, United Kingdom

Sam Muller, Regional Field Coordinator, Asia Regional HIV/AIDS Project, "An Australian Government Initiative", Ha Noi, Viet Nam

Roshan das Nair, Queen's Medical Centre, Nottingham, England

Dr. Russell Newcombe, Senior Lecturer in Drug Use & Addiction, School of Psychology, Faculty of Science, Liverpool John Moore's University, Liverpool, England

Dr Michael O'Dwyer, Senior Health Adviser, DFID South East Asia, Bangkok, Thailand.

Patrick O'Gorman, Consultant with Asian Harm Reduction Network and European Expert with UNDP led BUMAD and SCAD programmes, Nottingham, United Kingdom

James M. Oleske, MD, MPH, François-Xavier Bagnoud Professor of Pediatrics, Director, Division of Pulmonary, Allergy, Immunology & Infectious Diseases, Department of Pediatrics, New Jersey Medical School, Newark, NJ, Untied States

Hilgunn Olsen, SIRUS Norwegian Institute for Alcohol and Drug Research, Oslo, Norway

Kim Pate, Canadian Association of Elizabeth Fry Societies (CAEFS), Ottawa, Ontario, Canada

Mario Pecheny, Instituto Gino Germani, Universidad de Buenos Aires – CONICET, Buenos Aires, Argentina

Jia Ping, Beijing Aizhixing institute of health education, China

Dr.Hayley Pinto, Norfolk and Waveny Mental Health, Partnership NHS Trust, Norwich, Norfolk, United Kingdom

Robert Power PhD, Reader in Health & Social Sciences Research, Centre for Sexual Health & HIV Research, Department of Primary Care & Population Sciences, Royal Free & University College Medical School, Mortimer Market, London, United Kingdom

Dianne Proctor, Reproductive Health Activist, (Previously CEO of AHRA, now retired), Canberra, Australia

Kenn Quayle, i2i Peer Support, Gibsons, British Columbia, Canada

Pedro Ratis e Silva, DINAMO, São Paulo, Brazil

Melissa Raven, Lecturer, Coordinator, Drugs and Public Health, Department of Public Health, Flinders University, Bedford Park, Australia

Emran M. Razzaghi, M.D., M.P.H., Ass Professor of Psychiatry, Tehran University of Medical Sciences 2004-5 World Fellow, Yale University, New Haven, CT, USA

Thomas Timon Reichl, Bonn, Germany

Josiah D. Rich, M.D., M.P.H., Associate Professor of Medicine and Community Health, Brown University, Providence, RI, USA

Jane Richman, Substance Misuse Worker, United Kingdom

Kay Roberts, Glasgow, United Kingdom

Xavier Majó Roca, Programme on Drug Abuse, Department of Health. Autonomous, Government of Catalonia, Barcelona, Catalonia-Spain

Allan Rosenfield, MD, Mailman School of Public Health, Columbia University, New York, NY, USA

Rainer Rotthoff, Pelangi Communitry Foundation, Kuala Lumpur, Malaysia

Cliff Seaward, drug worker, United Kingdom

Gerard M. Schippers, Ph.D., Professor of Addictive Behaviors and Treatment Evaluation, Amsterdam Institute for Addiction Research (AIAR), Amsterdam, The Netherlands

Susan G. Sherman, PhD, MPH, Assistant Professor, Johns Hopkins Bloomberg School of Public Health, Baltimore, MD, USA

Ram Singh Gurung, an Ex-drug user, Advocacy coordinator, Naya Goreto, Kathmandu Nepal

Alfred Sommer, Dean and Professor, Johns Hopkins Bloomberg School of Public Health, Baltimore, MD, USA

Dr Mónica Suárez Cardona, Madrid, Spain

Brent Taylor, UNDUN, Kingston, Ontario, Canada

Dr. Gerald Thomas, Ottawa, Ontario, Canada

Juan Gabriel Tokatlian, Director Political Science and International Relations, Universidad de San Andres, Argentina

Bruce G. Trigg, MD, Medical Director, Sexually Transmitted Diseases Program, Department of Health, NM, USA

Pervaiz Tufail, Program Manager, AMAL Human Development Network, Islamabad, Pakistan

Azmi bin Uda, "Positive Muslim", Marang,Kuantan and Kuala Lumpur, Malaysia

Jamie Uhrig, Consultant in HIV Prevention and Care, Chiang Mai, Thailand

Kenneth A. Vail, M.P.H, M.A., Tenderloin AIDS Resource Center, San Francisco, CA, USA

Félix Vanderstricht, Aquadev NGO, Tools & Strategy Dpt., Brussels, Belgium

Camila Vega, Consultant, Regional Cooperative Mechanism to Monitor and Execute the ACCORD Plan of Action, United Nations Office on Drugs and Crime, Regional Centre for East Asia and the Pacific, Bangkok, Thailand

Miguel García Villanueva, Head of the Medical Services of the Prison of Pamplona, Spain

David Vlahov, Ph.D., New York, NY, USA

Jennifer Whittall, RhythmicPrinting, Victoria, British Columbia, Canada

Karen Willey, Fulcrum Medical Practice, Middlesbrough, England

Dr. Alex Wodak, Director, Alcohol and Drug Service, St. Vincent's Hospital, Sydney, Australia

Liron B. Wolff, LMSW, New York, NY, USA

Yang Yang, MPH, Yale University of Public Health, New Haven, CT, USA

Yuanyin, The Central University for Nationalities, China

Tomas Zabransky, M.D., Ph.D., Institute for Epidemiology, Public Health and Hygiene, Palacky University Olomouc, Czech Republic
