# Supplementary material for: Witch-hunt
Source: Harm Reduct J. 2005 Mar 4;2:3. doi: 10.1186/1477-7517-2-3 (PMC555579; doi:10.1186/1477-7517-2-3)
Supplement: Additional File 3 — Spanish version of the Open Letter [file 1477-7517-2-3-S3.pdf]

1 de marzo de 2005

Carta abierta a los delegados de la Sesión núm. 48 de la Comisión de Drogas Narcóticas (CND) de la ONU

En un año en el que la Oficina de las Naciones Unidas para las Drogas y el Crimen Organizado (UNODC) detenta la presidencia del órgano de gobierno del Programa Conjunto para el VIH/sida de la ONU (UNAIDS), nos dirigimos a Vds. para expresar nuestra preocupación ante los esfuerzos de los EE.UU. para obligar a la UNODC a retirar su apoyo a programas de recambio gratuito de jeringuillas, mantenimiento con metadona y otras medidas que se han demostrado eficaces a la hora de contener la expansión del VIH entre usuarios de drogas. El consumo de drogas por vía intravenosa es el principal canal de infección del VIH en docenas de países en Asia y la antigua Unión Soviética, incluyendo a Rusia, China, toda Asia central y la mayor parte del sudeste asiático. En la mayoría de países fuera de África, el mayor número de nuevas infectados corresponde a usuarios de drogas por vía intravenosa. Tal y como el director de la UNODC, Antonio Maria Costa, señaló en la Conferencia Internacional del Sida de julio de 2004, una respuesta eficaz a la epidemia de sida debida al consumo de drogas por vía intravenosa precisa ampliar los esfuerzos de prevención, incluyendo el recambio gratuito de jeringuillas, en lugar de políticas que aceleran el contagio del VIH a través del encarcelamiento ordinario e indiscriminado.

Desgraciadamente, los acontecimientos más recientes apuntan a que os EE.UU. están instando a la UNODC a que retire su apoyo a estrategias de prevención del VIH de demostrada eficacia precisamente en el momento en que sería necesario redoblar el apoyo a medidas como el recambio gratuito de jeringuillas y los tratamientos de sustitución con opiáceos. Consideramos particularmente alarmante que este esfuerzo por silenciar a la UNODC esté teniendo lugar en un año en el que la agencia detenta la presidencia del Comité de Organizaciones Copatrocinadoras de la UNAIDS, y en un año cuando la prevención del VIH es un foco temático de debate en la Sesión número 48 de la Comisión de Drogas Narcóticas (CND). Entre los hechos que han disparado nuestra alarma se encuentran:

§ El Sr. Costa, que el año pasado expresó públicamente su apoyo a cambios positivos en el código penal ruso, la expansión de programas de recambio gratuito de jeringuillas en países azotados por epidemias de sida debidas al consumo de drogas por vía intravenosa y otras medidas de reducción del daño provocado por el consumo de drogas, ha recibido una reprimenda desde el Departamento de Estado de los EE.UU. Tras una reunión con Robert Charles, Secretario Segundo de Asuntos Internacionales de Narcóticos y Aplicación de la Ley, el Sr. Costa se mostró dispuesto a revisar todas las referencias al concepto de "reducción del daño" de los documentos de la UNODC, impresos y electrónicos, y a estar "incluso más vigilante en el futuro".

§ En el sudeste asiático, la UNODC ha suspendido un programa que pretendía reducir la vulnerabilidad de los usuarios de drogas al VIH mediante un enfoque preventivo que ponía el énfasis en los aspectos de salud pública y de derechos humanos

de los usuarios de drogas, antes que en el castigo.

§ Incluso el recambio gratuito de jeringuillas, considerado una pieza no sólo eficaz sino esencial de la estrategia de prevención del VIH por parte de la UNAIDS, la OMS y muchas naciones miembros de la ONU, se ha vuelto inaceptable por cuestiones políticas. El pasado noviembre, un correo electrónico de un alto cargo de la UNODC dirigido a los empleados de menor rango pedía a estos últimos "asegurarse de que se evite toda referencia a la reducción del daño y el recambio gratuito de jeringuillas o agujas en los documentos, publicaciones y notas de prensa de la UNODC."

Admitimos que la UNODC depende de las contribuciones de las naciones donantes, y que los EE.UU. son el mayor donante a los programas de control de las drogas de la ONU. Pero, al mismo tiempo, las vidas de cientos de miles de personas dependen de enfoques científicos y sólidos al reto de la prevención del sida. Numerosos estudios, U.S. government studies, han revelado que estrategias como el recambio gratuito de jeringuillas y los tratamientos de sustitución con metadona reducen de manera demostrable la transmisión del VIH y otros riesgos para la salud. El hecho de que los delegados de los EE.UU. declaren que las evidencias científicas "no son convincentes", como hicieron en la sesión del año pasado de la Comisión de Drogas Narcóticas, no debería determinar el curso de los esfuerzos de la ONU en control de drogas y prevención del VIH, que se encuentran inextricable y esencialmente relacionados. Ni tampoco se debería pedir a la UNODC— agencia copatrocinadora de la UNAIDS, con un papel fundamental en la lucha contra la epidemia de VIH, que se abstenga de hacer declaraciones públicas a favor del recambio gratuito de jeringuillas sólo porque esta estrategia no cabe dentro de lo que los EE.UU. encuentran aceptable

Las estrategias que simplemente buscan fomentar la abstinencia de las drogas no son una alternativa aceptable a programas, tal como el recambio gratuito de jeringuillas, que ayudan a los consumidores de drogas en activo a protegerse del VIH y el sida. La experiencia ha demostrado que los esfuerzos de contención de las drogas basados en la "tolerancia cero" pueden tener el efecto de alejar a los usuarios de drogas por vía intravenosa de los tratamientos de desintoxicación y demás cuidados sanitarios. Esto es particularmente cierto en muchos países donde los esfuerzos de control del mercado de estupefacientes conducen a falsos arrestos, agresiones físicas y extorsiones por parte de la policía, detención prolongada sin juicio, curas de desintoxicación obligatorias, penas de cárcel desproporcionas y en condiciones crueles, y, en algunos casos, ejecuciones extrajudiciales. Programas tales como el recambio gratuito de jeringuillas y los tratamientos substitución con opiáceos, en cambio, previenen nuevas infecciones de VIH y además constituyen un puente hacia otros tratamientos sanitarios. Restringir la aplicación de estos programas es un flagrante violación del derecho humano a la salud de los usuarios de drogas.

Con todo nuestro respeto, les urgimos a que, cuando este año se reúnan a debatir sobre la prevención del VIH y el abuso de drogas, expresen su apoyo a los programas de recambio gratuito de jeringuillas, los tratamientos de sustitución con opiáceos y otros enfoques basados en el principio de reducción del daño de demostrada eficacia para reducir el

riesgo de contagio del VIH, que defiendan el derecho humano a la salud de los usuarios de drogas, y que planten cara a los esfuerzos para dejar de lado las evidencias científicas y maniatar a aquellos que trabajan combatiendo el VIH y el sida al pie del cañón. Lo que está en juego, nada menos, es el futuro de la epidemia de VIH.

cc: Programa Conjunto para el VIH/SIDA de las Naciones Unidas Organización
cc: Mundial de la Salud
cc: Oficina del Alto Comisionado para Derechos Humanos
cc: Junta Internacional de Control de Narcóticos

Las siguientes organizaciones e individuos han firmado esta carta del primero de marzo de 2005:

Organizaciones por Región

Asia

AIZHIXING Institute of Health Education, Beijing, China

Asian Harm Reduction Network (AHRN), Chiang Mai, Thailand

Asia Pacific Rainbow, New Delhi, India

Australian Drug Foundation, Melbourne, Australia

Australian Injecting and Illicit Drug Users League, Darlinghurst, Australia

Blue Diamond Society, Kathmandu, Nepal

Burnet Institute, Melbourne, Australia and Yangon, Myanmar

The Centre for Harm Reduction, Macfarlane Burnet Institute for Medical Research & Public Health, Melbourne, Australia

Community Health, Rehabilitation, Education & Awareness (CREA), Dhaka, Bangladesh

Drug Action Committee of the City of Greater Geelong, Victoria, Australia

Family Drug Support, Willoughby, Australia

Health and Development Networks (HDN), Chiang Mai, Thailand

ILGLAW – Asia, New Delhi, India

Lawyers Collective HIV/AIDS Unit, New Delhi, India

Malaysian AIDS Council, Kuala Lumpur, Malaysia

Malaysian Harm Reduction Working Group, Kuala Lumpur, Malaysia

Northern Sydney Central Coast Health, Sydney, Australia

Pinoy Plus Association, Manila, Philippines

RISE, Peshawar, Pakistan

Thai AIDS Treatment Action Group (TTAG), Bangkok, Thailand

Thai Drug Users' Network (TDN), Bangkok, Thailand

Turning Point Alcohol and Drug Centre, Melbourne, Australia

VIVAIDS Inc., Fitzroy, Australia

WartaAIDS, Jakarta, Indonesia

Western Australian Substance Users Association, Bunbury, Australia

Yayasan Spiritia, Jakarta, Indonesia

África

AfriCASO: African Council of Aids Service Organization, Dakar, Senegal

Kenya AIDS NGOs Consortium (KANCO), Nairobi, Kenya

Sheryl's Orphans Children Home, Nairobi, Kenya

UNDP/Kinshasa, Kinshasa, Democratic Republic of Congo

Europa/Asia Central

Abraço—Associação de Apoio a Pessoas Infectadas e Afectadas pelo VIH /SIDA, Lisbon, Portugal

Act Up—Paris, Paris, France

Action Against AIDS Germany, Tubingen, Germany

Actions Traitements, Paris, France

AGIHAS (PLWHA Support group), Riga, Latvia

AIDES NGO, Pantin Cedex, France

AIDS Action Europe, Amsterdam, Netherlands

AIDS Foundation East-West (AFEW), Moscow, Russia

Aids-Hilfe Bonn e. V., Bonn, Germany

AKZEPT E.V.—Bundesverband für akzeptierende Drogenarbeit und humane Drogenpolitik, Berlin, Germany

Amsterdam Institute for Addiction Research (AIAR), Amsterdam, Netherlands

A.N.O., Prague, Czech Republic

Associazione Mastropietro & Co., Turin, Italy

Associazione Nazionale Italiana Lotta AIDS (A.L.A.), Milano, Italy

Blupoint Drug Counselling Centre, Budapest, Hungary

Bremen Institute for Drug Research, Bremen, Germany

CA Odyseus, Bratislava, Slovakia

Center for Interdisciplinary Research on Women and Gender (ZFG), Oldenburg, Germany

Central and Eastern European Harm Reduction Network, Vilnius, Lithuania

The Centre for Research on Drugs and Health Behaviour, Imperial College, United Kingdom

Charitable Foundation "Rehabilitation Center of Drug Addicts "Virtus", Dnipropetrovsk, Ukraine

The Chrysalis Drug Project, Hertford, United Kingdom

Coalition on Vulnerable Population "I Can Live", Vilnius, Lithuania

Community Organization of People Living with HIV & AIDS, Moscow, Russia

Convictus Eesti, Tallinn, Estonia

Cranstoun Drug Services, London, United Kingdom

DIA+LOGS NGO, Support centre for those affected by HIV/AIDS, Riga, Latvia

Die Deutsche Gesellschaft für Suchtmedizin (vorm DGDS) e.V., Hamburg, Germany

DroBeL - Drogenberatung Lehrte e. V., Lehrte, Germany

Drogenberatung e.V., Bielefeld, Germany

Drogprevenciós Alapítvány, Budapest, Hungary

Dublin AIDS Alliance Ltd., Dublin, Ireland

Equal to Equal, Almaty, Kazakhstan

Estima Associação, Leiria, Portugal

Északi Támpont Egyesület, Budapest, Hungary

European AIDS Treatment Group, Brussels, Belgium

European Coalition for Just and Effective Drug Policies (ENCOD), Antwerp, Belgium

European Commission, Coordination of Anti-drugs Policy, Directorate-General Justice, Freedom and Security, Brussels, Belgium

Evangelisches Stadtjugendpfarramt Gera, Gera, Germany

Förderverein interdisziplinärer Sucht- und Drogen-forschung (FISD) e.V., Zentrum für interdisziplinäre Sucht-und drogen-forschung – ZIS, Hamburg, Germany

Freundes- und Förderkreis Suchtkrankenhilfe e.V., Wuppertal, Germany

GAT - Grupo Português de Activistas sobre Tratamentos de VIH/SIDA, Lisbon, Portugal

George House Trust, Manchester, United Kingdom

Grazia Zuffa, Fuoriluogo/Il Manifesto, Rome, Italy

Greater Glasgow Drug Action Team, Glasgow, Scotland

Grup Igia, Barcelona, Spain

Grupo de Trabajo sobre Tratamientos del VIH (gTt), Barcelona, Spain

Health and Social Development Foundation, Sofia, Bulgaria

Health Education Association NGO, Yerevan, Armenia

Helsinki Foundation for Human Rights, Warsaw, Poland

Hemp Seed Association, Budapest, Hungary

HIV Prevention Programme at Kekava, Riga, Latvia

Hope-Sofia, Sofia, Bulgaria

Indro e.V., Münster, Germany

Initiative for Health Foundation, Sofia, Bulgaria

The Initiative of Drug Users' Mutual Support, Vilnius, Lithuania

The Institute for Criminal Policy Research (ICPR), School of Law, King's College, London, United Kingdom

Institut für Sozialpädagogik, Technische Universität Berlin, Berlin, Germany

The International Community of Women Living with HIV/AIDS (ICW), London, United Kingdom

International Harm Reduction Association, London, United Kingdom

Irish Penal Reform Trust, Dublin, Ireland

Itaca Europe, Rome, Italy

Italian League for the fight against AIDS - Center for Human Rights and Public Health (LILA CEDIUS ONLUS), Milan, Italy

JES e.V., Bielefeld and Bremen, Germany

John Mordaunt Trust, London, United Kingdom

Mainline Foundation, Amsterdam, Netherlands

Malopolskie Stowarzyszenie PROBACJA (PROBACJA Association), Krakow, Poland

MARATON, Warsaw, Poland

Methadone Alliance, London, United Kingdom

MODUS VIVENDI, Prévention du sida et réduction des risques pour usagers de drogues, Brussels, Belgium

Monar Krakow Drugs Project, Krakow, Poland

MONAR, Pulawy, Poland

National AIDS Trust, London, United Kingdom

Netherlands Drug Policy Foundation (SDB), Haarlem, Netherlands

NGO TRUST, Skopje, Macedonia

Osservatorio Italiano sull'Azione Globale contro l'AIDS, Rome, Italy

PASSAGE, Skopje, Macedonia

Plymouth Drug and Alcohol Action Team, Plymouth, United Kingdom

Quest for Quality, Amsterdam, Netherlands

REFORM, Drug Policy Interest Group, Essex, United Kingdom

RFSU, The Swedish Association for Sexuality Education, Stockholm, Sweden

Romanian Association against AIDS (ARAS), Bucharest, Romania

Romanian Harm Reduction Network (RHRN), Bucharest, Romania

Russian Harm Reduction Network, Moscow, Russia

SANANIM, Prague, Czech Republic

Sensoa (Flemish Centre for Expertise and Services on Sexual Health and HIV), Antwerp, Belgium

SIDACTION – International Programs, Paris, France

SIDA STUDI, Barcelona, Spain

SignPost Forth Valley, Stirling, Scotland

Social AIDS Committee, Warsaw, Poland

SOMA – Associação Portuguesa Anti-proibicionista, Lisbon, Portugal

Steunpunt Druggebruikers, Antwerp, Belgium

T3E [UK] Ltd., London, United Kingdom

Transform Drug Policy Foundation, Bristol, United Kingdom

Transnational Institute (TNI) Drug and Democracy Programme, Amsterdam, Netherlands

Tver AIDS Center, Tver, Russia

Tver Center of Drugs Addiction Treatment, Tver, Russia

United Kingdom Harm Reduction Alliance Eccles, Kent, United Kingdom

Women for Women's Human Rights (WWHR) - New Ways, Istanbul, Turkey

World AIDS Campaign, Amsterdam, The Netherlands

Zentrum für interdisziplinäre Frauen- und Geschlechterforschung – ZFG, Carl von Ossietzky Universität Oldenburg, Oldenburg, Germany

Zentrum Suchtmedizin Klinikum Wahrendorff, Sehnde, Germany

Americas

Argentinean Harm Reduction Association (ARDA), Buenos Aires, Argentina

Argentinean Harm Reduction Network (REDARD), Buenos Aires, Argentina

Asociación Civil "Convivencia" de Personas Viviendo y Conviviendo con VIH/SIDA, Buenos Aires, Argentina

Brazilian Drug Users Network, Recife, Brazil

Fundación para Estudio e Investigación de la Mujer. Buenos Aires, Argentina

HUMANAR, Huritaba, Brazil

Intercambios Asociación Civil, Buenos Aires, Argentina

International Women Aids Caucus, Buenos Aires, Argentina

LACCASO - Latin American and Caribbean Council of AIDS Service Organizations, Caracas, Venezuela

Mujer y Salud en Uruguay (MYSU), Montevideo, Uruguay

ONG INTERPARES, Parana Entre Rios, Argentina

Parana Drug Users Network, Curitiba, Brazil

Programa Alter-Acciones, El Abrojo – Instituto de Educación Popular, Montevideo, Uruguay

REDUC, Brazilian Harm Reduction Network, São Paulo, Brazil

RELARD, Latin American Harm Reduction Network, Curitiba, Brazil

Medio Oriente/África del Norte

Iranian National Center for Addiction Studies (INCAS), Tehran, Iran

The Moroccan Association for the Fight Against AIDS, Marrakesh, Morocco

Persepolis Harm Reduction NGO, Tehran, Iran

Persia+, UNDP GIPA Programme, Tehran, Iran

Norte America

Access Works! Harm Reduction Services, Minneapolis, MN, USA

Act Up—East Bay, Oakland, CA, USA

Advocates for Recovery Through Medicine, Connecticut Chapter, New London, CT, USA

After Hours Project, Inc., Brooklyn, NY, USA

AIDS Foundation of Chicago, Chicago, IL, USA

AIDS Project Los Angeles, Los Angeles, CA, USA

AIDS Treatment Data Network, New York, NY, USA

American Academy of HIV Medicine, Washington DC, USA

American Foundation for AIDS Research, Washington DC, USA

ARC International, Ottawa, Ontario, Canada

Canadian Harm Reduction Network, Toronto, Ontario, Canada

Canadian HIV/AIDS Legal Network, Montreal, Quebec, Canada

Center for Health and Gender Equity (CHANGE), Takoma Park, MD, USA

Center for Human Rights and Public Health, Johns Hopkins Bloomberg School of Public Health, Baltimore, MD, USA

CHAMP (Community HIV/AIDS Mobilization Project), New York, NY, USA

Chicago Recovery Alliance, Chicago, IL, USA

CitiWide Harm Reduction, Bronx, NY, USA

Drug Overdose Prevention & Education Project, San Francisco, CA, USA

DrugSense, Irvine, CA, USA

Exponents, New York, NY, USA

Foundation for Integrative AIDS Research (FIAR), Brooklyn, NY, USA

Gay Men's Health Crisis, New York, NY, USA

Global AIDS Alliance, Washington DC, USA

Harm Reduction Coalition, New York, NY, USA

Harm Reduction Project, Denver and Salt Lake City, UT, USA

Health GAP (Global Access Project), New York, NY, USA

HIV Advocacy Council of Oregon and SW Washington, Portland, OR, USA

HIV Resource Center, Roseburg Risk Reduction, Roseburg, OR, USA

Housing Works, Inc., New York, NY, USA

Human Rights Watch, New York, NY, USA

International Antiprohibitionist League, New York, NY, USA and Rome, Italy

International Center for Advancement of Addiction Treatment, Baron Edmond de Rothschild Chemical Dependency Institute of Beth Israel Medical Center, New York, NY, USA

International Council of AIDS Service Organizations (ICASO), Toronto, Ontario, Canada

International Foundation for Alternative Research in AIDS (IFARA), Portland, OR, USA

International Gay and Lesbian Human Rights Commission, New York, NY, USA

International Women's Health Coalition, New York, NY, USA

Lower East Side Harm Reduction Center, New York, NY, USA

Montefiore Medical Center, Division of Public Health and Policy Research, Bronx, NY, USA

NAMA-Norcal, Santa Cruz, CA, USA

National Association of People with AIDS (NAPWA), Silver Spring, MD, USA

National Association for Victims of Transfusion-Acquired AIDS, Bethesda, MD, USA

The New York Academy of Medicine, New York, NY, USA

Open Society Institute, New York, NY, USA

Physicians for Human Rights, Washington, DC, USA

Positive Health Project, Inc., New York, NY, USA

Prevention Point Pittsburgh, Pittsburgh, PA, USA

TAG, Treatment Action Group, New York, NY, USA

Unified Networkers of Drug Users Nationally, Kingston, Ontario, Canada

Firmas Individuales

Vittorio Agnoletto, Scientific Mgr of LILA CEDIUS, Member of the European Parliament, GUE/NGL Group, Italy

Waheed Ahmad, Advocate High Court, Legal Secretary: World Asian Workers Organisation, Lahore, Pakistan

Joan Anderson, HIV/AIDS community Consultant and volunteer in Toronto, Ontario, Canada

Guillermo R. Aureano, Ph.D., Chercheur associé, Groupe d'étude et de recherche sur la sécurité internationale (GERSI), Département de science politique, Université de Montréal, Montréal, Quebec, Canada

Brambillaschi Barbara, L.I.L.A. - Lega Italiana per la Lotta contro l'AIDS-sede di Como, Italy

Trude Bennett, The University of North Carolina at Chapel Hill, School of Public Health, Chapel Hill, NC, USA

Dennis Berg, Ph.D., Professor of Sociology, California State University, Fullerton, CA, USA

Marie-Andrée Bertrand, Professor emeritus, University of Montreal, Criminology, Montréal, Quebec, Canada

Suzanne Bessoir, Harm Reduction Specialist, AIDS Service Center NYC, New York, NY, USA

Calum Blair, Harm Reduction worker, SignPost Forth Valley, Stirling, Scotland

Simon Boerboom, Psychiatrist, Amsterdam, Netherlands

Beth M. Bouloukos, Ithaca, New York, NY, USA

Scott Burris, James E. Beasley Professor of Law, Temple University Beasley School of Law, Philadelphia, PA, USA

Ryan Borgen, Student Global AIDS Campaign, Northeastern University School of Law, Boston, MD, USA

Jill Britton, London, United Kingdom

Damon Brogan, Manager, VIVAIDS, Melbourne, Australia

Benjamin Bynum, Fikelela AIDS Project, Cape Town, South Africa

Vincenzo Caracciolo, Associazione P24 Lila Livorno, Livorno, Italy

Craig Carmichael, The University of Queensland, Brisbane, Australia

Paul Causey, HIV/AIDS Program Consultant, Bangkok, Thailand

Jennifer Chapman, MPH, Project Manager/Research Coordinator, Center for Clinical, Epidemiology & Biostatistics, University of Pennsylvania, School of Medicine, Philadelphia, PA, USA

George K. Clarke, Volunteer CT Director, CMA, New London, CT, USA

Shelley Cogger, Research Assistant, Post Release Experience of Prisoners in Queensland (PREP-Q), Queensland Alcohol and Drug, Research and Education Centre (QADREC), University of Queensland, Australia

Franco Corleone, Forum Droghe, Rome, Italy

Melissa Dent, Community Development Worker, Melbourne, Australia

Tamara Desiatov, Koondoola, Western Australia

Kate Dickie, Annah Pickering, Managers, Auckland Branch of the New Zealand Prostitutes Collective, Auckland, New Zealand

Dr. Gyaw Htet Doe, Senior Consultant Psychiatrist, Taunggyi Drug Treatment Hospital, Taunggyi, Myanmar

Ms J Dowling, Auburn, NSW, Australia

Andy Dudley, Addaction, Derby, United Kingdom

Carolina Pecheny Durozier, Paris, France

Brian R. Edlin, M.D., Associate Professor of Medicine and Public Health, Center for the Study of Hepatitis C, Weill Medical College of Cornell University, New York, NY, USA

Maria Fotopoulou, Ph.D. student, Imperial College, University of London, London, United Kingdom

Mayada Youssef Fox, staff member, HIV/AIDS Department, WHO HQ, Geneva, Switzerland

Sandra Fox, former staff, Harm Reduction sector, Melbourne, Australia

Jonathan Freedlander, Baltimore, MD, USA

Liliana Gherman, MD, Program Director, Public Health Program, Soros Foundation, Chisinau, Moldova

Cees Goos, honorary consultant Anton Proksch Institut, former WHO staff, Vienna, Austria

Chris W. Green, Jakarta, Indonesia

Heath Greville (individual support), Department of Health, Perth, Western Australia

Patrick Griffiths, PhD candidate, Contemporary Globalisation and HIV/AIDS in Vietnam, RMIT University, Melbourne, Australia

Revd Dr Ian T Guy, General Practitioner, Fulcrum Medical Practice, Middlesbrough, United Kingdom

Bianca L. Guzman, Ph.D., CHOICES Director of Research, La Puente, CA, USA

Fiona Hale, International Network Manager, ICW, London, United Kingdom

Jen Hall, Information and Referral (Duty) Worker, Inner South Community Health Service, Prahran, Australia

Helena Hansen, MD-Ph.D. Candidate, Yale University School of Medicine, New Haven, CT, USA

Paul Hardacre, Training Officer, Asian Harm Reduction Network (AHRN), Chiang Mai, Thailand

Frank Harding, Ayer's Cliff, Quebec, Canada

Andy Hart, Vendafit Pty Ltd, Sydney, Australia

Paul J. von Hartmann, Project P.E.A.C.E., Planet Ecology Advancing Conscious Economics, Santa Rosa, CA, USA

Robert Heimer, Ph. D., Associate Professor, Department of Epidemiology and Public Health Center for Interdisciplinary Research on AIDS Yale University School of, New Haven, CT, USA

Beatriz Acevedo Holguin, Researcher on International Drug Policy, Hull, United Kingdom

Lital Hollander, Research Manager, ESMAN Medical Consulting, Milano, Italy

Danny Holness, Peer Education Officer, RaveSafe, Victoria, Australia

Daniel E. Hood, Ph.D., Department of Criminal Justice/Security Systems, State University of New York at Farmingdale, Farmingdale, New York, NY, USA

Annabelle Horton, UN Office of Drugs and Crime, Bangkok, Thailand.

Neil Hunt, Senior Research Associate, University of Kent; Honorary Research Fellow, Centre for Research on Drugs and Health Behaviour, Imperial College, London; Director, UK Harm Reduction Alliance Eccles, Kent, United Kingdom

Elena Jeffreys, Scarlet Alliance, Australias Sex Worker Association, Canberra Act, Australia

McKinzie McClay Jernberg, Minneapolis, MN, USA

Debbie Johnson, Macclesfield, Cheshire, United Kingdom

P. R. W. Kendall, MBBS, MSc, FRCPC, Provincial Health Officer, Ministry of Health Services, Victoria, British Columbia, Canada

Joseph Kim, Young Drug Users Peer Education Officer, VIVAIDS (Victorian Drug User Organisation), Melbourne, Australia

Dr Stuart A. Kinner, Lecturer, Queensland Alcohol and Drug, Research and Education Centre (QADREC), Queensland, Australia

Debbie Kocziban, Addaction, Plymouth Devon, United Kingdom

Nicky Kupfer, Alcohol Tobacco and other drugs service, Sydney, Australia

Heather La Faye LPN, Medicine cures sickness,all the world is medicine, what is the self?, Recovery Resource Center, Minneapolis, MN, USA

Susana Lambrechts, Esteban Echeverría, Argentina

Deb Lapthorne, Director of Public Health, Plymouth Primary Care Trust/ Plymouth City Council, Plymouth, United Kingdom

Fiona Leibrick, Lecturer: Health Sciences (Alcohol & Other Drug Studies) / Registered Psychologist, Charles Darwin University, Darwin, Australia

Sarah Lippek, AIDS Center of Queens County, Long Island City, NY, USA

Deirdre Love, Senior Health Promotion Specialist HIV Positive People, Health First, London, United Kingdom

Wendy Loxley, Associate Professor, National Drug Research Institute, Curtin University of Technology, Bentley, West Australia

Devon MacFarlane, Vancouver, British Columbia, Canada

Catherine Mackenzie, St. Catharines, Ontario, Canada

Jan Chrostek Maj MD, Rydygier's Hospital, Krakow, Poland

Paolo La Marca, Project manager (Harm reduction & training), LILA CEDIUS (Italian league fight against AIDS), Milano, Italy

David Martin, Program Medical Officer, Health Canada, Vancouver, British Columbia, Canada

Phyra M. McCandless, Johns Hopkins School of Public Health, Baltimore, MD, USA

Rachel McLean, MPH Candidate, Johns Hopkins University Bloomberg School of Public Health, Baltimore, MD, USA

Belinda McNair, Senior Project Officer-City Drug Safety Plan, City of Melbourne, Melbourne, Australia

Cristina Menoyo, Secretaría del Plan Nacional sobre el Sida, Ministerio de Sanidad y Consumo, Madrid. España

Maria Luisa Milesi, Esteban Echeverría, Argentina

Dr Peter Miller, Senior Clinical Research Worker. National Addiction Centre (Maudsley Hospital/Institute of Psychiatry). King's College London, London, United Kingdom

PhDr. Michal Miovsky, Ph.D., Institute of psychology, Academy of Sciences of the Czech Republic, Prague, Czech Republic

Andrew Moss Ph.D, Professor of Epidemiology and Medicine, University of California, San Francisco, CA, USA

Marjorie "Mo" Mowlam, Kent, United Kingdom

Sam Muller, Regional Field Coordinator, Asia Regional HIV/AIDS Project, "An Australian Government Initiative", Ha Noi, Viet Nam

Roshan das Nair, Queen's Medical Centre, Nottingham, England

Dr. Russell Newcombe, Senior Lecturer in Drug Use & Addiction, School of Psychology, Faculty of Science, Liverpool John Moore's University, Liverpool, England

Dr Michael O'Dwyer, Senior Health Adviser, DFID South East Asia, Bangkok, Thailand.

Patrick O'Gorman, Consultant with Asian Harm Reduction Network and European Expert with UNDP led BUMAD and SCAD programmes, Nottingham, United Kingdom

James M. Oleske, MD, MPH, François-Xavier Bagnoud Professor of Pediatrics, Director, Division of Pulmonary, Allergy, Immunology & Infectious Diseases, Department of Pediatrics, New Jersey Medical School, Newark, NJ, Untied States

Hilgunn Olsen, SIRUS Norwegian Institute for Alcohol and Drug Research, Oslo, Norway

Kim Pate, Canadian Association of Elizabeth Fry Societies (CAEFS), Ottawa, Ontario, Canada

Mario Pecheny, Instituto Gino Germani, Universidad de Buenos Aires – CONICET, Buenos Aires, Argentina

Jia Ping, Beijing Aizhixing institute of health education, China

Dr.Hayley Pinto, Norfolk and Waveny Mental Health, Partnership NHS Trust, Norwich, Norfolk, United Kingdom

Robert Power PhD, Reader in Health & Social Sciences Research, Centre for Sexual Health & HIV Research, Department of Primary Care & Population Sciences, Royal Free & University College Medical School, Mortimer Market, London, United Kingdom

Dianne Proctor, Reproductive Health Activist, (Previously CEO of AHRA, now retired), Canberra, Australia

Kenn Quayle, i2i Peer Support, Gibsons, British Columbia, Canada

Pedro Ratis e Silva, DINAMO, São Paulo, Brazil

Melissa Raven, Lecturer, Coordinator, Drugs and Public Health, Department of Public Health, Flinders University, Bedford Park, Australia

Emran M. Razzaghi, M.D., M.P.H., Ass Professor of Psychiatry, Tehran University of Medical Sciences 2004-5 World Fellow, Yale University, New Haven, CT, USA

Thomas Timon Reichl, Bonn, Germany

Josiah D. Rich, M.D., M.P.H., Associate Professor of Medicine and Community Health, Brown University, Providence, RI, USA

Jane Richman, Substance Misuse Worker, United Kingdom

Kay Roberts, Glasgow, United Kingdom

Xavier Majó Roca, Programme on Drug Abuse, Department of Health. Autonomous, Government of Catalonia, Barcelona, Catalonia-Spain

Allan Rosenfield, MD, Mailman School of Public Health, Columbia University, New York, NY, USA

Rainer Rotthoff, Pelangi Communitry Foundation, Kuala Lumpur, Malaysia

Cliff Seaward, drug worker, United Kingdom

Gerard M. Schippers, Ph.D., Professor of Addictive Behaviors and Treatment Evaluation, Amsterdam Institute for Addiction Research (AIAR), Amsterdam, The Netherlands

Susan G. Sherman, PhD, MPH, Assistant Professor, Johns Hopkins Bloomberg School of Public Health, Baltimore, MD, USA

Ram Singh Gurung, an Ex-drug user, Advocacy coordinator, Naya Goreto, Kathmandu Nepal

Alfred Sommer, Dean and Professor, Johns Hopkins Bloomberg School of Public Health, Baltimore, MD, USA

Dr Mónica Suárez Cardona, Madrid, Spain

Brent Taylor, UNDUN, Kingston, Ontario, Canada

Dr. Gerald Thomas, Ottawa, Ontario, Canada

Juan Gabriel Tokatlian, Director Political Science and International Relations, Universidad de San Andres, Argentina

Bruce G. Trigg, MD, Medical Director, Sexually Transmitted Diseases Program, Department of Health, NM, USA

Pervaiz Tufail, Program Manager, AMAL Human Development Network, Islamabad, Pakistan

Azmi bin Uda, "Positive Muslim", Marang,Kuantan and Kuala Lumpur, Malaysia

Jamie Uhrig, Consultant in HIV Prevention and Care, Chiang Mai, Thailand

Kenneth A. Vail, M.P.H, M.A., Tenderloin AIDS Resource Center, San Francisco, CA, USA

Félix Vanderstricht, Aquadev NGO, Tools & Strategy Dpt., Brussels, Belgium

Camila Vega, Consultant, Regional Cooperative Mechanism to Monitor and Execute the ACCORD Plan of Action, United Nations Office on Drugs and Crime, Regional Centre for East Asia and the Pacific, Bangkok, Thailand

Miguel García Villanueva, Head of the Medical Services of the Prison of Pamplona, Spain

David Vlahov, Ph.D., New York, NY, USA

Jennifer Whittall, RhythmicPrinting, Victoria, British Columbia, Canada

Karen Willey, Fulcrum Medical Practice, Middlesbrough, England

Dr. Alex Wodak, Director, Alcohol and Drug Service, St. Vincent's Hospital, Sydney, Australia

Liron B. Wolff, LMSW, New York, NY, USA

Yang Yang, MPH, Yale University of Public Health, New Haven, CT, USA

Yuanyin, The Central University for Nationalities, China

Tomas Zabransky, M.D., Ph.D., Institute for Epidemiology, Public Health and Hygiene, Palacky University Olomouc, Czech Republic
